# Active-site protein dynamics and solvent accessibility in native *Achromobacter cycloclastes* copper nitrite reductase

**DOI:** 10.1107/S2052252517007527

**Published:** 2017-06-16

**Authors:** Kakali Sen, Sam Horrell, Demet Kekilli, Chin W. Yong, Thomas W. Keal, Hakan Atakisi, David W. Moreau, Robert E. Thorne, Michael A. Hough, Richard W. Strange

**Affiliations:** a School of Biological Sciences, University of Essex, Wivenhoe Park, Colchester CO4 3SQ, England; b Scientific Computing Department, STFC Daresbury Laboratory, Warrington WA4 4AD, England; c Physics Department, Cornell University, Ithaca, NY 14853, USA

**Keywords:** serial crystallography, high temperature, catalysis, molecular dynamics, density functional theory, denitrification, copper nitrite reductase, radiolysis, synchrotron radiation

## Abstract

Microbial nitrite reductases are denitrifying enzymes that are a major component of the global nitrogen cycle. Multiple structures measured from one crystal (MSOX data) of copper nitrite reductase at 240 K, together with molecular-dynamics simulations, have revealed protein dynamics at the type 2 copper site that are significant for its catalytic properties and for the entry and exit of solvent or ligands to and from the active site. Molecular-dynamics simulations were performed using different protonation states of the key catalytic residues (Asp_CAT_ and His_CAT_) involved in the nitrite-reduction mechanism of this enzyme. Taken together, the crystal structures and simulations show that the Asp_CAT_ protonation state strongly influences the active-site solvent accessibility, while the dynamics of the active-site ‘capping residue’ (Ile_CAT_), a determinant of ligand binding, are influenced both by temperature and by the protonation state of Asp_CAT_. A previously unobserved conformation of Ile_CAT_ is seen in the elevated temperature series compared with 100 K structures. DFT calculations also show that the loss of a bound water ligand at the active site during the MSOX series is consistent with reduction of the type 2 Cu atom.

## Abstract 2

Multiple structures obtained from one crystal of copper nitrite reductase at elevated cryogenic temperature, together with molecular-dynamics simulations, reveal catalyically important protein and solvent dynamics at the active site.

## 1. Introduction   

Copper nitrite reductases (CuNiRs) are key catalytic enzymes in the denitrification pathway of the global nitrogen cycle (Zumft, 1997 ▸). They are homotrimeric proteins built from monomeric subunits consisting of two cupredoxin-like domains enclosing a type 1 copper electron-transfer site (T1Cu) and a catalytic type 2 copper site (T2Cu). The two Cu atoms are separated by a Cys–His electron-transfer bridge spanning ∼12.5 Å. The T2Cu binds nitrite and catalyses its conversion to nitric oxide *via* a one-electron reduction in a reaction that requires two protons: NO_2_^−^ + 2H^+^ + e^−^ ↔ NO + H_2_O (Eady & Hasnain, 2003 ▸; Brenner *et al.*, 2009 ▸). In the resting-state structure previously reported at 0.9 Å resolution (Antonyuk *et al.*, 2005 ▸; Adman *et al.*, 1995 ▸), the T2Cu is coordinated by three histidine residues (His100, His135 and His306) and a water molecule, with the latter being displaced when nitrite is bound. The T2Cu is located between adjacent monomers ∼12 Å from the protein surface. Solvent, nitrite or other small molecules such as formate, acetate, nitrous oxide and azide (Tocheva *et al.*, 2008 ▸) may enter the active-site pocket through channels connected to the bulk solvent. The normal catalytic product, NO, is thought to exit through these same channels. An isoleucine residue (Ile_CAT_) that caps the active-site pocket has been proposed to provide steric constraints to ligand access and selectivity to ligand binding (Boulanger & Murphy, 2003 ▸; Tocheva *et al.*, 2008 ▸), while critical aspartate (Asp_CAT_) and histidine (His_CAT_) residues are required for the correct ligand positioning, hydrogen bonding and proton delivery during catalysis (Antonyuk *et al.*, 2005 ▸; Boulanger *et al.*, 2000 ▸; Boulanger & Murphy, 2001 ▸). In *Achromobacter cycloclastes* nitrite reductase (*Ac*NiR) these are the Ile257, Asp98 and His255 residues.

Crystal structures of *Ac*NiR at cryogenic temperatures revealed that two alternative positions of Asp98 are possible: a ‘gatekeeper’ position and a ‘proximal’ position. In the proximal position, Asp98 forms a hydrogen-bond interaction with His255 *via* a bridging water molecule, while this interaction is lost in the gatekeeper position. In cryogenic structures, His255 has been reported to be largely unperturbed by the proximal-to-gatekeeper shift of Asp98 and the associated change in the hydrogen-bonding network. However, in room-temperature (RT) XFEL and synchrotron-radiation structures of *Alcaligenes faecalis* NiR (*Af*NiR), changes in the hydrogen-bonding network of His_CAT_ with the neighbouring Glu and Thr residues are presented as key steps in the CuNiR reaction mechanism (Fukuda *et al.*, 2016 ▸). The active-site ‘capping residue’ Ile257, along with Leu308, Val142 and Ala137, contributes to the shaping of a solvent channel identified as one of the operative channels for proton and nitrite delivery to the T2Cu and for exit of NO product from the active site.

Protein dynamics vary significantly with crystal temperature (Frauenfelder *et al.*, 2009 ▸; Halle, 2004 ▸), and increasingly temperature is becoming a useful variable in crystallography to study the relationship between dynamics and function (Keedy *et al.*, 2015 ▸). Typically, X-ray crystallographic data are measured from crystals maintained at 100 K in order to immobilize X-ray-generated free radicals and damaged protein and minimize radiation damage, leading an increase in crystal lifetime of up to two orders of magnitude relative to RT (Southworth-Davies *et al.*, 2007 ▸). Between RT and 100 K, protein crystals undergo at least one and possibly several temperature-dependent transitions (Weik & Colletier, 2010 ▸; Lewandowski *et al.*, 2015 ▸; Ringe & Petsko, 2003 ▸; Keedy *et al.*, 2015 ▸). Notably, anharmonic macromolecular motions resume above the glass transition that occurs in the range 180–220 K, where solvent viscosity is greatly reduced. Maintaining crystal order and diffraction quality in the range between this transition temperature and RT is challenging, but recently experimental advances have opened this regime for study. In this work, we have obtained a crystal structure of wild-type *Ac*NiR at the standard cryogenic temperature of 100 K and a series of structures from one crystal (MSOX; Horrell *et al.*, 2016 ▸) at 240 K, a temperature that allows anharmonic motion while still extending the resolution and crystal lifetime beyond those achievable at RT (Warkentin & Thorne, 2010 ▸). In addition, we have examined the active-site protein dynamics and solvent accessibility using all-atom molecular dynamics and DFT calculations based on the crystal structures. The 240 K data reveal a new alternative T2Cu active-site conformation and, together with simulations, shows how active-site water structure correlates with the protonation states of active-site residues. Our data provide insights into the dynamic motion of *Ac*NiR beyond that which may be gained from static, single-crystal structures determined at 100 K.

## 2. Methods   

### 2.1. Protein purification and crystallization   

Wild-type *Ac*NiR was expressed and purified as described previously (Antonyuk *et al.*, 2005 ▸). *Ac*NiR crystals were grown in space group *P*2_1_3 by hanging-drop vapour diffusion against 100 m*M* sodium citrate pH 5.0, ∼1.7 *M* ammonium sulfate. The crystals were cryocooled by plunging them into liquid nitrogen. For data collection at both 240 and 100 K, no additional penetrating cryoprotective agents beyond those present in the mother liquor were used.

### 2.2. Crystallographic data collection, processing and refinement   

A series of structures were measured at 240 K from one wild-type *Ac*NiR crystal on beamline F1A at MacCHESS. The crystal was mounted on a polymer loop, covered with a polymer capillary containing reservoir solution at one end to prevent dehydration, and then placed on the goniometer in a nitrogen-gas stream at 240 K. The crystal was illuminated with a 100 µm beam, which was larger than the maximum crystal dimension of ∼75 µm, and a total of seven MSOX data sets were recorded from the same illuminated crystal volume. Experiments were performed with an X-ray wavelength of 0.97 Å and with an incident photon flux of 2.3 × 10^10^ s^−1^ using a PILATUS 6M detector (Kraft *et al.*, 2009 ▸). Each data set in the MSOX series, comprising a total of 80 images, was obtained with 0.5° oscillation and 0.5 s exposure per image. To compare the 240 K data series with a standard cryogenic data set, a single data set from an *Ac*NiR crystal was measured at 100 K on beamline I02 at Diamond Light Source employing a PILATUS 6M-F (Dectris) detector (Kraft *et al.*, 2009 ▸). Experiments were performed with an X-ray wavelength of 0.98 Å and with beam dimensions of 24 × 24 µm. The complete data set was collected using 0.1° oscillation and 0.1 s exposure per image. The total X-ray dose per data set was estimated using *RADDOSE*-3*D* (Zeldin *et al.*, 2013 ▸).

Data sets were processed using *XDS* (Kabsch, 2010 ▸) and *AIMLESS* (Evans & Murshudov, 2013 ▸), with a CC_1/2_ ≥ 0.5 cutoff (Karplus & Diederichs, 2012 ▸) and *I*/σ(*I*) ≥ 1 in the outermost resolution shell. Merging and refinement statistics are given in Table 1 ▸. Structures were refined using *REFMAC*5 (Murshudov *et al.*, 2011 ▸), with 5% of the data excluded to calculate the free *R* factor (Brünger, 1992 ▸). For the 240 K MSOX data, anisotropic temperature factors were used in the refinement of the initial 1.38 Å resolution data set, and isotropic refinement was used for subsequent data sets with lower resolution owing to radiation damage. The starting model was taken from the 0.9 Å resolution wild-type *Ac*NiR structure (PDB entry 2bw4; Antonyuk *et al.*, 2005 ▸). Manual remodelling of the structure and addition of water molecules in the 2*F*_c_ − *F*_o_ and *F*_c_ − *F*_o_ electron-density maps was accomplished iteratively using *Coot* (Emsley *et al.*, 2010 ▸) and *REFMAC*. The copper-ligand sites were unrestrained during refinement. The stereochemistry of the final models and the goodness of fit to the electron-density maps were assessed using *Coot*, *MolProbity* (Chen *et al.*, 2010 ▸) and *JCSG Quality Control Check*. All regions of the structure were well ordered except for a short segment of a flexible loop between residues Lys194 and Ala202 and at the N- and C-terminal ends, where the electron density was weak. Figures were rendered using *PyMOL* v.1.8 (Schrödinger).

### 2.3. Quantum chemical calculations   

Minimum cluster models of the T2Cu site were derived from the MSOX series of data sets. These consisted of Cu(His)_3_ and the active-site residues Asp98, His255 and Ile257 that are implicated either in proton transfer or in substrate binding, along with the two or one coordinating water molecules. These clusters were subjected to DFT calculations to understand the electronic structure of the T2Cu under the influence of the electrostatic and steric effects of the two important active-site residues, Asp98 and Ile257, respectively. The optimizations were carried out for both the copper(I) and copper(II) oxidation states for two experimentally observed conformations of Ile_CAT_, with Asp_CAT_ in its protonated state. The Asp_CAT_ and His_CAT_ residues were protonated in accordance with the low pH used in crystallization. Furthermore, the spectroscopic and DFT studies of nitrite-bound T2Cu of Ghosh and coworkers are consistent with protonated Asp_CAT_ and His_CAT_ at low pH (Ghosh *et al.*, 2009 ▸). All residues were truncated at the C^α^ atoms, which were fixed at their crystallographic positions, and the valency was adjusted by the addition of H atoms. The B3LYP functional was used for the optimization together with the DFT-D3 dispersion correction (Grimme *et al.*, 2010 ▸). The def2-TZVP basis was used for the Cu atoms and def2-SVP for the other atoms (Weigend & Ahlrichs, 2005 ▸). The optimizations were carried out using the DL-FIND geometry optimization library (Kästner *et al.*, 2009 ▸) in *ChemShell* (Sherwood *et al.*, 2003 ▸) interfaced to the *ORCA* package (Neese, 2012 ▸) for DFT calculations.

### 2.4. Molecular dynamics   

The crystal structures reported here at 100 and 240 K both have two water molecules bound to the T2Cu in the initial MSOX data set. Wild-type *Ac*NiR structures at 100 K have also been reported with a single coordinated water molecule (Antonyuk *et al.*, 2005 ▸). We have explored the MD at 293 K using both of these coordination spheres for the starting structures, examining the solvent accessibility at the T2Cu for different protonation states of key active-site ligands. The coordinates of the wild-type *Ac*NiR monomer with one coordinated water were taken from the 0.9 Å resolution crystal structure PDB entry 2bw4 (Antonyuk *et al.*, 2005 ▸) and the data set with two bound waters was taken from this study. In both cases the homotrimeric biological unit was generated by symmetry operations. Utilizing the *propKa* module of the *PDB*2*PQR* suite of programs (Dolinsky *et al.*, 2004 ▸), followed by visual inspection of the local side-chain environments, the protonation states of the titratable residues were adjusted to be consistent with pH 5, the pH condition used for the crystallization of *Ac*NiR. Previous work from Ghosh *et al.* (2009 ▸) supported the protonation of both Asp_CAT_ and His_CAT_ at pH 5.0 and the protonation of only His_CAT_ at pH 7. His_CAT_ at pH 5 is considered here and the alternative protonation states examined by MD are considered in the Supporting Information, including deprotonated His_CAT_ states, Asp98p-HSE (histidine residue singly protonated at N^∊^) and Asp98p-HSD (histidine residue singly protonated at N^δ^). The empirical p*K*_a_ value for the aspartate acid side chain is 3.9 and, depending on the microenvironment of the protein active site, Asp_CAT_ could maintain a dynamic equilibrium between its protonated and deprotonated states at pH 5. Accordingly, two protonated systems were prepared to mimic the possible active-site microenvironment at pH 5, in one of which both Asp_CAT_ and His_CAT_ are protonated, while in the other His_CAT_ is protonated and Asp_CAT_ is deprotonated. These systems are referred to henceforth in this manuscript as ‘Asp98p’ and ‘Asp98’. After adjustment of the protonation states, the overall charges of the Asp98p and Asp98 systems were +6 and +3, respectively.

Model complexes to represent the T1Cu and T2Cu, [Cu^II^(Imz)_2_(CH_3_CH_2_S(CH_3_))(CH_3_S^−^)] and [Cu^II^(Imz)_3_(H_2_O)], respectively, were optimized at the MP2 level to derive the partial charges on the Cu ions. These were derived from the electrostatically fitted Merz–Kollman potential using a van der Waals radius of 2.0 Å for copper (Sigfridsson & Ryde, 1998 ▸), to yield formal partial electronic charges of +0.46 (T1Cu) and +1.1135 (T2Cu). The coordination sphere and geometry around the copper ions were fixed to the crystal structure and the molecular-mechanics parameters were adapted from the CHARMM36 force-field database (Best *et al.*, 2012 ▸).

The systems were solvated with a 15 Å layer of TIP3P water (Jorgensen *et al.*, 1983 ▸). Chloride counter-ions were added in order to maintain the electroneutrality of the simulation models. Explicit all-atom MD simulations were performed on these systems using *NAMD* 2.9 (Phillips *et al.*, 2005 ▸) with the CHARMM36 force field. These simulations employed Langevin dynamics with periodic boundary conditions at 293 K. Long-range electrostatics were treated by the particle mesh Ewald method. In the NPT simulations the pressure was maintained with the Langevin piston method. Both systems were initially subjected to 5000 steps of conjugate-gradient (CG) minimization to eliminate any unphysical contacts. Next, the water and ions were equilibrated in an NVT ensemble, keeping the protein fixed for 1 ns. This was followed by 5000 steps of CG minimization and 5 ns equilibration under the NPT ensemble, keeping the backbone harmonically restrained (5 kcal^−1^ mol^−1^ Å^2^) and the coordination spheres of both the T1Cu and T2Cu sites [Cu(His)_2_(Met)(Cys^−^) and Cu(His)_3_(H_2_O), respectively] constrained at their crystallo­graphic coordinates. The simulation was continued for another 50 ns after removing the backbone restraints. During the sampling runs, with the exception of the water coordinated to the T2Cu, all of the constraints on the ligands at the T1Cu and T2Cu were maintained. The trajectories from MD were analysed using *VMD* (Humphrey *et al.*, 1996 ▸)

## 3. Results and discussion   

### 3.1. MSOX structures of wild-type *Ac*NiR at 240 K   

**Figure 1:**
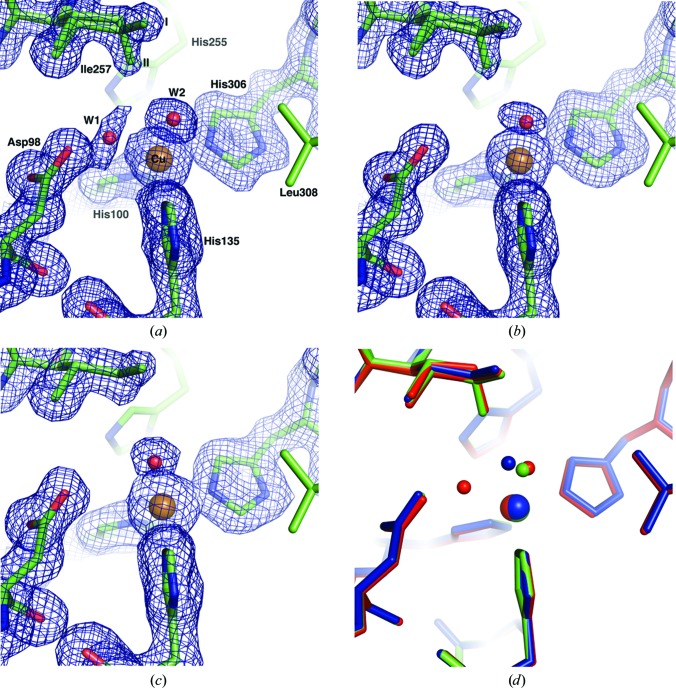
Sequential data sets for wild-type *Ac*NiR at 240 K, showing X-ray-induced reorganization of bound water molecules and the orientation of Ile257 at the T2Cu. The Cu ligands, Ile257 and proximal Asp98 residues are modelled in 2*F*_o_ − *F*_c_ electron-density maps contoured in the range 0.53–0.43 e Å^−3^. (*a*) ds1_240K_, the initial data set at 1.38 Å resolution, with two bound water molecules (W1 and W2) and two conformations (I and II) of the Ile257 side chain. Asp98 forms a ∼2.1 Å hydrogen bond to W1. (*b*) ds2_240K_ at 1.47 Å resolution, with the W1 site vacated, leaving one bound water W2 and with both Ile257 conformations I and II present. (*c*) ds3_240K_ at 1.65 Å resolution, with W2 and Ile257 present only in conformation I. (*d*) Comparison of the ds1_240K_ (red), ds2_240K_ (green) and ds3_240K_ (blue) serial structures, showing ‘migration’ of W2 into the centre of the T2Cu cavity, occupying in ds3_240K_ the space freed by the absence of Ile257 conformation II.

Three structures were selected from the 240 K MSOX series, during which the data resolution declined from 1.38 Å in the first data set (ds1_240K_) to 1.65 Å in the final selected data set (ds3_240K_). Superposition of the C^α^ atoms of these structures with the 0.9 Å resolution wild-type *Ac*NiR structure (PDB entry 2bw4; Antonyuk *et al.*, 2005 ▸) gave an r.m.s.d. of 0.1 Å. The catalytic T2Cu of ds1_240K_ is shown in Fig. 1 ▸(*a*). The Cu atom (*B* factor of 9.3 Å^2^) is coordinated to three histidine residues (His100, His135 and His306) at 2.03–2.08 Å and two water molecules (W1 and W2) at ∼2.0 and 2.12 Å. The extended electon density between Asp98 and the T2Cu suggests that W1 adopts a range of positions in the crystal. This is not observed at 100 K (see below). The catalytically important Asp98 and His255 residues are linked by hydrogen bonds to a bridging water molecule, while the Asp98 side chain is present in a single (proximal) conformation. The electron-density map is consistent with there being two conformations of the Ile257 side chain present in the crystal, each initially modelled with 0.5 occupancy according to their *B* factors. In conformation I the Ile257 CD1 atom is positioned as previously reported for wild-type and ligand-bound *Ac*NiR structures obtained at 100 K (Antonyuk *et al.*, 2005 ▸; Horrell *et al.*, 2016 ▸), while in the alternate conformation II it is oriented towards the type 2 Cu atom, shortening its separation from the type 2 Cu atom by 1.5 Å. Conformation II has only previously been observed in a structure of ascorbate-reduced *A. faecalis* CuNiR (PDB entry 1aq8; Murphy *et al.*, 1997 ▸). This arrangement of the Ile257 side chain effectively compresses the solvent- or ligand-accessible volume of the active-site cavity, with the distance from the Ile257 CD1 atom to W2 being reduced from 3.4 Å in conformation I to 2.62 Å in conformation II. Of the two bound water molecules, W1 lies between the type 2 Cu atom and the Asp98 side chain and appears to be more labile (*B* factor of 34.1 Å^2^) than W2 (*B* factor of 18.8 Å^2^), which suggests partial occupancy for this water molecule. Upon further X-ray exposure of the crystal during the data series (ds2_240K_, resolution 1.47 Å) one water is lost from the T2Cu coordination sphere, leaving a four-coordinate copper site with three His ligands at an average distance of 2.04 Å and W2 (*B* factor of 32.9 Å^2^) at 2.13 Å (Fig. 1 ▸ *b*). By the final data set in the series (ds3_240K_, resolution 1.65 Å), the *B* factors of the Cu atom and W2 have increased to 13.0 and 36.7 Å^2^, respectively, and the Cu–W2 distance to 2.20 Å. The average Cu(His)_3_ distance is 2.06 Å. A significant structural change observed between ds2_240K_ and ds3_240K_ is that the Ile257 residue is present in ds3_240K_ in conformation I only with full occupancy (Fig. 1 ▸ *c*), while W2 shifts its position into the space that is made available by this change (Fig. 1 ▸ *d*).

### 3.2. The structure of wild-type *Ac*NiR at 100 K   

A single structure (ds1_100K_) of the wild-type protein was obtained at 1.4 Å resolution at the standard macromolecular cryogenic temperature of 100 K (Table 1 ▸). The T2Cu has a similar coordination to that observed at the higher temperature, with two coordinated water molecules at 2.01 Å and 2.23 Å with *B* factors of 22 and 21 Å^2^, respectively. In contrast to the initial structure that was obtained in the 240 K series, the Ile257 side chain was found to be present only in conformation I (Supplementary Fig. S1). This result is in line with the previous crystal structures measured at 100 K.

### 3.3. T2Cu water coordination   

The present crystallographic data reveal that two waters are coordinated to the T2Cu atom, and in the 240 K MSOX series one of the coordinated waters is lost from the T2Cu following X-ray exposure. Previous 100 K crystal structures reported only one water coordinated to the T2Cu in *Ac*NiR (Antonyuk *et al.*, 2005 ▸). One hypothesis to explain the change in coordination at the T2Cu with X-ray dose is reduction of the T2Cu atom. Previously, we have shown that the T1Cu is rapidly reduced in nitrite-bound *Ac*NiR crystals prepared and measured at 100 K (Horrell *et al.*, 2016 ▸), while reduction of wild-type T2Cu has also been observed (Fukuda *et al.*, 2016 ▸). This supports the possibility of electron transfer from the T1Cu to the T2Cu occurring between the collection of data sets ds1_240K_ and ds3_240K_ in the series.

**Figure 2:**
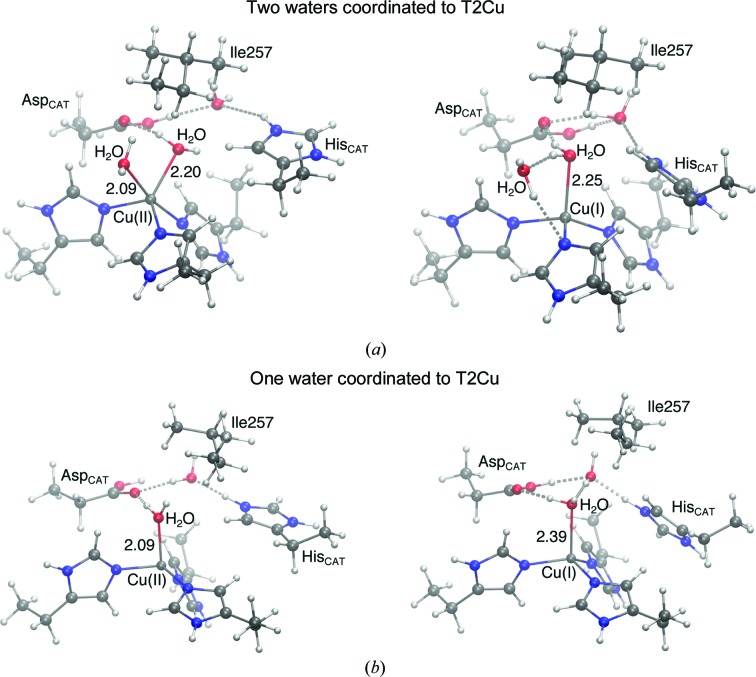
DFT-optimized structures of the T2Cu with different oxidation states, modelled from crystal structures obtained at 240 K. (*a*) Oxidized state (left) showing two waters coordinated and the reduced state (right) with one water lost from the coordination sphere. (*b*) Oxidized state (left) with one water coordinated and the reduced state (right) in which this water is retained with an increased bond length. Both Asp_CAT_ and His_CAT_ are protonated and distances are given in Å.

Quantum chemical studies lend support to this hypothesis. The structure with two waters coordinated to the T2Cu is consistent with a copper(II) state. Upon reduction to copper(I), one of the coordinated waters is lost and the T2Cu geometry changes from pentacoordinate to tetracoordinate, an observation consistent with the MSOX data (Fig. 2 ▸ *a*). The loss of one water on the reduction of T2Cu is independent of the protonation of either O atom of Asp_CAT_. The optimized structures shown in Fig. 2 ▸ most closely correspond to the initial crystal structure. The structures with the alternate Asp_CAT_ oxygen-protonation state are given in Supplementary Fig. S2. In the presence of only one water, the T2Cu(II) structure is optimized to give a tetracoordinate site with one water and three His residues bound to T2Cu. The average water–copper distance is ∼2.1 Å. When the T2Cu atom is reduced there is no loss of the bound water and the structure converges to a tetracoordinate site with a lengthening of the copper–water distance by ∼0.3 Å (Fig. 2 ▸ *b*). Protonation of either of the O atoms of Asp_CAT_ results in elongation of the Cu—H_2_O bond on reduction of the T2Cu (Supplementary Fig. S2). The coordination sphere of T2Cu appears to be unaffected by the conformation of Ile257 in these DFT models.

### 3.4. The protonation state of Asp_CAT_ influences the solvent accessibility of T2Cu   

Low-temperature crystal structures revealed that the Asp_CAT_ residue can adopt two positions within the active site: ‘proximal’ and ‘gatekeeper’. It is hypothesized that in the gatekeeper position Asp_CAT_ facilitates proton and substrate delivery along the solvent channels that link the T2Cu to the bulk surface and that the proximal position is chemically relevant for nitrite reduction to occur. MD simulations were undertaken to study the alternative conformations of Asp_CAT_ and the solvent accessibility at the active T2Cu site. Details referring to the one-water simulations are given here, with additional data in the Supporting Information for the two-water case, which gives essentially the same results. The overall trimeric structure of the protein is found to be preserved throughout the all-atom MD simulations, with an overall r.m.s.d. of <1.8 Å. The r.m.s.d. for the protein heavy atoms within a 10 Å sphere of the T2Cu is <0.9 Å.

**Figure 3:**
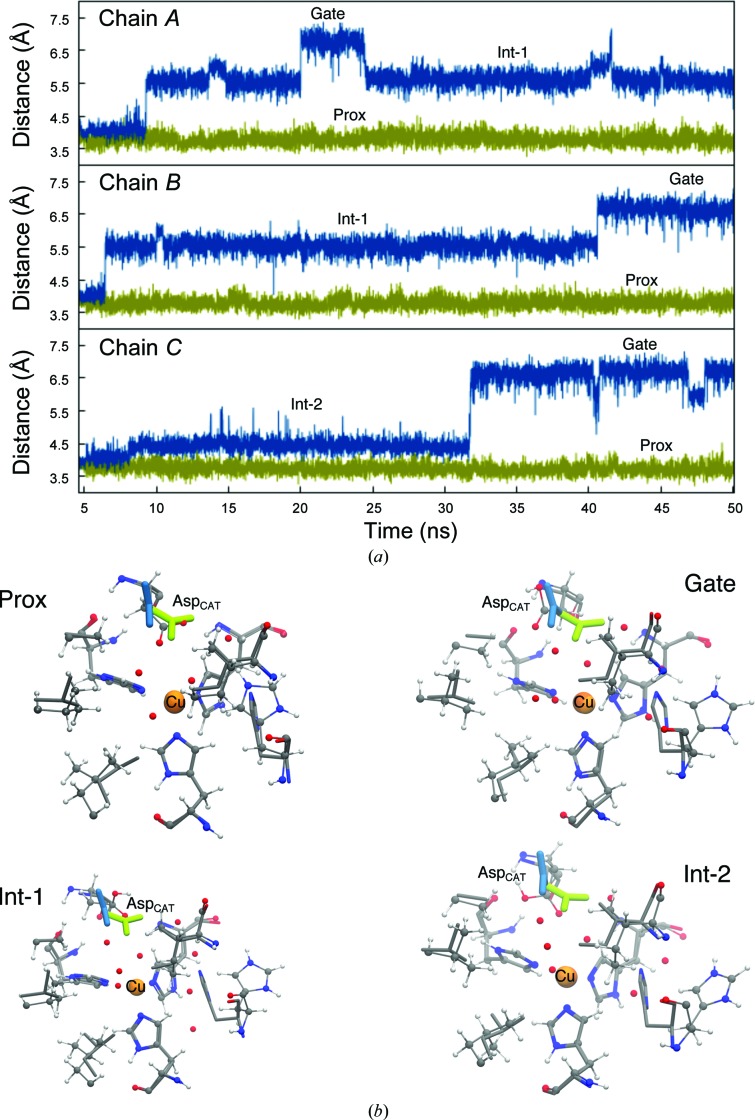
Time evolution of the centre-of-mass distance between the T2Cu and the Asp98 residue in its protonated and unprotonated states. (*a*) The MD trajectories for the Asp98p (blue) and Asp98 (green) states are shown for the three monomers of the *Ac*NiR trimer. The separation between centres of mass for Asp98 and the type 2 Cu atom remains constant at ∼3.8 Å throughout the simulation. In Asp98p the separation increases in all three monomers to ∼6–7 Å within 7–9 ns in chains *A* and *B* and by 31 ns in chain *C*. This increased distance is associated with a switch of the Asp98p residue between its proximal and gatekeeper orientations, a motion that is not observed in the Asp98 protein over the same timescale. Asp98p shows three distinct positions in the MD trajectories, one short-lived and only observed in the initial part of the simulation, which corresponds to the proximal position of Asp98, a second gatekeeper configuration, as observed in cryogenic structures, and a third at intermediate positions (Int-1 and Int-2) to the gatekeeper position, as shown by MD snapshots after ∼20 ns in (*b*). The MD simulation is shown in ball-and-stick representation and the crystal structure by thin lines. The two conformations of Asp_CAT_ in the proximal and gatekeeper positions are shown in thicker green and blue lines, respectively. His_CAT_ is displaced from its crystallographic position in the MD when Asp98 is not protonated (‘prox’), but it remains hydrogen-bonded to the bridging water. In the Int-1, Int-2 and gate positions, His_CAT_ has rotated away from the crystallographic position and no longer forms the bridging hydrogen bond.

To track the existence of the two Asp_CAT_ positions observed in the experimental low-temperature crystal structures, the centre-of-mass distance between the carboxylate group of Asp_CAT_ and the T2Cu from MD is shown in Fig. 3 ▸. For the Asp98p system, the proximal orientation is observed only in the initial 5–10 ns, followed by a transition to the gatekeeper position *via* intermediate orientations. The maximum deviation of the Cu–Asp98p (centre of mass) distance is 6.8 ± 0.2 Å, which corroborates with the 5.8 Å Cu–Asp_CAT_ distance observed for the gatekeeper position in the low-temperature crystal structure. In the Asp98 system, only the proximal position of Asp_CAT_ is observed. The corresponding Cu–Asp_CAT_ distance is 3.8 ± 0.2 Å, which is in good agreement with that observed in the crystal structure (4.2 Å).

**Figure 4:**
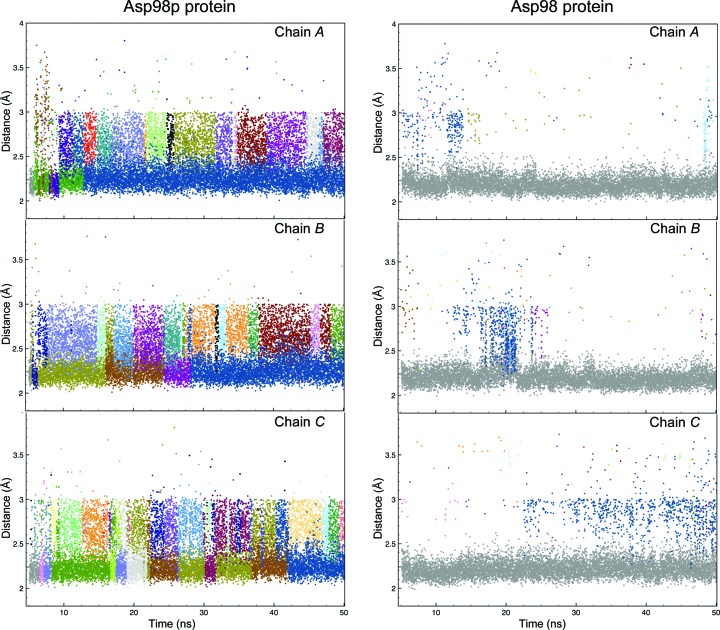
Solvent accessibility at the active-site pocket during MD simulations. Water molecules within 3.0 Å of the type 2 Cu atom in each monomer (chains *A*, *B* and *C*) of the *Ac*NiR trimer are shown in different colours, with the bound water in the original crystal structure shown in grey. The enhanced access and increased solvent exchange at the T2Cu for the three monomers is evident in Asp98p (left panels) compared with the relatively sparse solvent population in deprotonated Asp98 (right panels).

An estimate of water accessibility was obtained by counting the number of water molecules within a 3 Å sphere of the type 2 Cu atom in each monomer of the *Ac*NiR trimer. Fig. 4 ▸ shows the solvent accessibility at the T2Cu active-site pocket during the dynamics, and clearly shows enhanced exchange of water in the Asp98p protein. Two or more water molecules in each monomer of the Asp98p protein trimer are present within the 3 Å sphere around the type 2 Cu atom for 75 ± 5% of the 50 ns MD simulations, a proportion which reduced nearly tenfold to 8 ± 3% when the protein was in the Asp98 deprotonated state (Supplementary Fig. S3). MD shows that Asp_CAT_ remains in the proximal position when in the deprotonated Asp98 state, regardless of the protonation state of the His_CAT_ (Fig. 3 ▸ and Supplementary Fig. S4). The extremely low exchange of water for the Asp98 system could arise from a strong electrostatic interaction between the Asp_CAT_ and His_CAT_ residues, assisted *via* a water molecule restricting the space around T2Cu for effective water exchange (see Fig. 6*a*). On the other hand, when Asp_CAT_ is protonated, its interaction with His_CAT_ is weakened, thereby promoting the transition of Asp_CAT_ from the proximal to the gatekeeper position and thus providing room for water exchange and enhancing water accessibility in the water channel. CuNiRs require the efficient transfer of two protons to the T2Cu site to effectively reduce nitrite to nitric oxide. Within the microenvironment of the protein, these accessible water molecules along with polar and ionic amino acids can facilitate such proton transfers.

**Figure 5:**
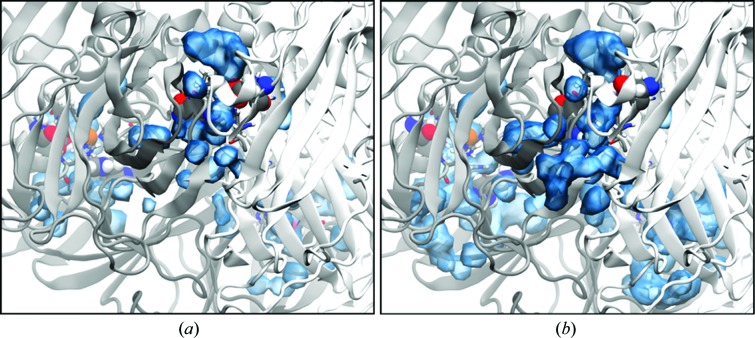
Volumetric maps of solvent content at the T2Cu during MD simulations. The figure shows the frame-averaged water occupancy over the 50 ns trajectory for (*a*) Asp98 and (*b*) Asp98p. The enhanced mobility and the shift in the position of the His_CAT_ and Asp_CAT_ residues in the Asp98p protein increases the access of solvent flowing through the inter-domain cleft to the T2Cu. This is also a feasible route for the entry or exit of the substrate and product during catalysis.

The appreciable increase in the number and throughput of exchangeable water molecules occupying the active-site pocket in Asp98p (Fig. 5 ▸) is not owing to an overall increase in the volume of the hydrophobic channel (defined by Val142, Ala137, Leu308 and Ile257), but appears to be triggered by the switch of the Asp98p side chain from the proximal to the gatekeeper position. This movement does not occur when Asp_CAT_ is deprotonated (Fig. 3 ▸), but does occur for all systems where Asp_CAT_ is protonated, irrespective of the protonation state of His_CAT_. This includes the alternative deprotonated His_CAT_ states Asp98p-HSE and Asp98p-HSD, which also showed enhanced water accessibility and exchange at the T2Cu active site (Supplementary Figs. S4, S5 and S6) and hydrophobic channels (Supplementary Figs. S7 and S8).

**Figure 6:**
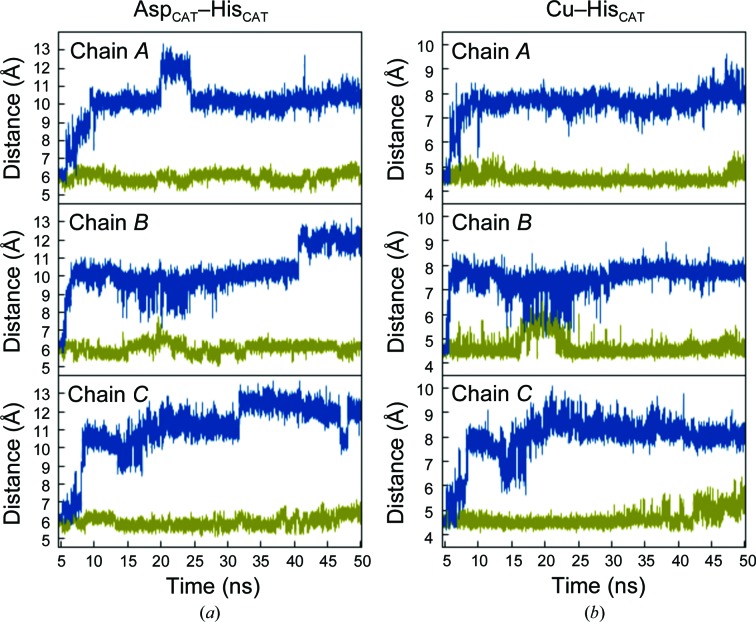
Time evolution of the His_CAT_ residue in each monomer of the *Ac*NiR trimer during MD simulations. (*a*) Distances between the centre of mass of His_CAT_ ring atoms (heavy atoms only) and the centre of mass of the Asp98p carboxylic group (blue) and Asp98 (green) states. (*b*) Distance between the centre of mass of His_CAT_ atoms and the T2Cu.

The dynamic behaviour of the His_CAT_ residue is strongly correlated to the motions of Asp_CAT_. Specifically, the deprotonated Asp98 residue is constrained in the proximal position by hydrogen bonding, *via* a bridging water, to the protonated His255. The Asp98–His255 separation is maintained at ∼6 Å throughout the MD simulation. In the protonated state, Asp98p is no longer held by this hydrogen-bonding network and can adopt the alternative gatekeeper position, while the His255 residue is also less constrained and is able to adopt a different geometry, rotating to a position further away from the T2Cu (Fig. 6 ▸). Several representative structures of the displaced His_CAT_ with its immediate surrounding environment are given in Supplementary Fig. S9. In 53 ± 4% of MD trajectory snapshots, His_CAT_ is engaged in the formation of either one or two hydrogen bonds to bulk water (Supplementary Fig. S10). In these concerted movements of the active-site residues, the separation between the type 2 Cu atom and His255 increases within the first 5 ns of the simulation from ∼4.5 to ∼7.5 Å, while the Asp98p–His255 distance increases to 10–12 Å. This occurs independently for each monomer. Asp98p-HSE MD shows a similar trend for His_CAT_ movement. However, in the Asp98p-HSD simulation the His_CAT_ residue remains stabilized in its crystallographic position *via* a hydrogen bond to the backbone O atom of Glu279.

Experimentally, both the proximal and gatekeeper positions of Asp_CAT_ have been observed in *Ac*NiR (and in other CuNiR) crystal structures (Antonyuk *et al.*, 2005 ▸; Horrell *et al.*, 2016 ▸), while the ‘pH effect’ on the Asp_CAT_–W–His_CAT_ bridge that we describe here has yet to be observed in crystals. His_CAT_ has been proposed to adopt alternative conformations related to its proton-transfer role, based upon a combined XFEL and synchrotron study of a related CuNiR from *A. faecalis* (Fukuda *et al.*, 2016 ▸). We note that only one conformation is observed in all of the crystal structures presented here, albeit in synchrotron structures with relatively high X-ray doses.

Considering the protonation state of Asp_CAT_, the experimentally observed conformations are both visible in the MD simulations and are highly dependent on its protonation state. In the deprotonated state the proximal conformation dominates, while protonation allows Asp_CAT_ to adopt the gatekeeper position, independent of the protonation state of His_CAT_. Experimentally, Asp_CAT_ is in the deprotonated state at pH ∼6, the optimum pH for CuNiR reduction in *Af*NiR (Zhang *et al.*, 2000 ▸; Kataoka *et al.*, 2000 ▸; Kakutani *et al.*, 1981 ▸). Protonation of Asp_CAT_ at low pH is consistent with spectroscopic and DFT studies by Ghosh *et al.* (2009 ▸). The MD simulations of the native enzyme show that a dynamic equilibrium of Asp_CAT_ in its protonated and deprotonated forms is highly feasible, with the balance between the two states being controlled by the active-site pH. Protons may be provided by either the bulk water in the active site, as represented in our MD by the Asp98p system, or from His_CAT_ *via* the bridging water molecule, as represented by the Asp98p-HSE and Asp98p-HSD MD systems (see Supporting Information).

If His_CAT_ is the proton source for Asp_CAT_ protonation, this would correspond to the Asp98p-HSD MD system. Here, the His_CAT_ position is maintained close to the initial crystal geometry (Supplementary Fig. S11). Loss of the proton could also lead to the Asp98p-HSE system, which behaves similarly to the Asp98p system, where His_CAT_ is not the proton donor. In these systems His_CAT_ adopts a geometry that is not observed experimentally. A closer inspection of the protein structure reveal that this His_CAT_ is located in the inter-domain region and is potentially part of a channel from the active site to the bulk solvent. Hence, movement of this residue occurs without perturbing the overall structure of the protein, and alters the hydrogen-bond interactions with the water molecules in the channel (Supplementary Fig. S10)

### 3.5. Dynamics of the active-site ‘capping residue’ Ile_CAT_ are influenced by the temperature and the Asp_CAT_ protonation state   

**Figure 7:**
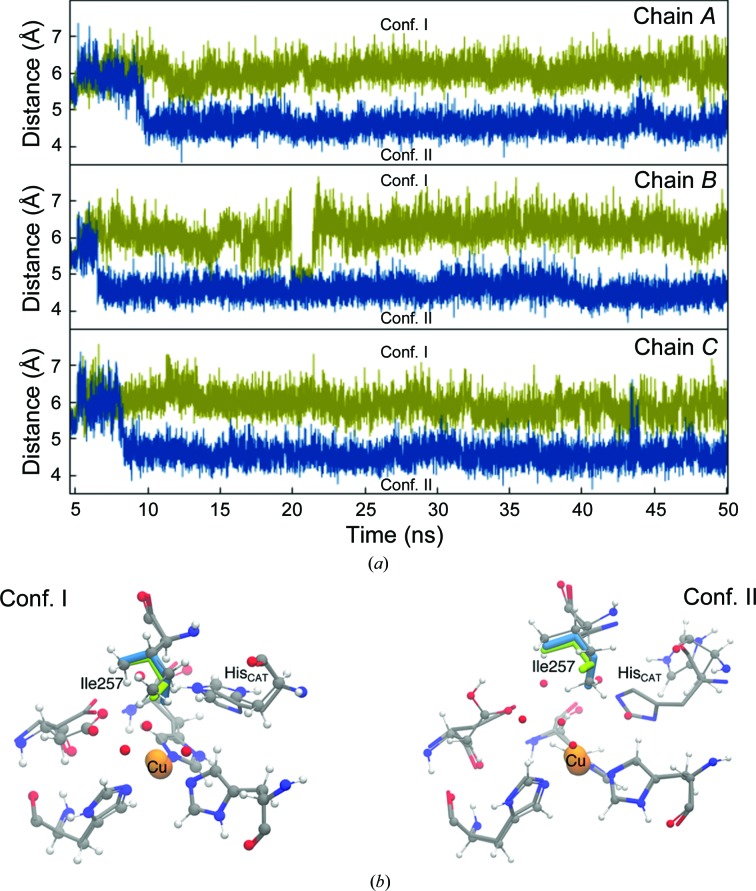
Time evolution of the Ile257 residue in each monomer of the *Ac*NiR trimer during MD simulations. (*a*) The distance between the sterically important Ile257 residue side-chain CD1 atom and the type 2 Cu atom is shown for the protonated Asp98p (blue) and deprotonated Asp98 (green) states of the protein. In Asp98p, the Ile257 CD1 side-chain atom is preferentially oriented towards the type 2 Cu atom in conformation II, compressing the space between them and in a position that would impose limits on ligand selectivity and binding geometry at the active site. In its deprotonated Asp98 state, Ile257 is predominantly found in the conformation I position, although fluctuations between conformations I and II may also occur, as seen around ∼20 ns for monomer *B* of the *Ac*NiR trimer (middle panel). (*b*) shows the position of Ile257 in conformation I (right) and conformation II (left) overlaid with the crystal structure. In (*b*), the MD conformation is shown in ball-and-stick representation and the crystal structure by thin lines. The two conformations of Ile257 in conformations I and II are shown in thicker green and blue lines, respectively.

The structures show a clear temperature dependence of the dynamics of the Ile_CAT_ residue, which exists in two possible orientations in the crystal at 240 K, with one of them being inhibited at 100 K (Fig. 1 ▸). These conformational fluctuations of Ile_CAT_ are the first to be observed in the crystalline state and would likely have greater freedom to occur in the solution state. This is confirmed by the molecular-dynamics simulations performed at 293 K. The MD also suggest that the orientation of the Ile_CAT_ residue is strongly influenced by the protonation state of Asp_CAT_ and the reorientation of His_CAT_ (Fig. 7 ▸; also see Supporting Information). In the deprotonated state, Ile257 preferentially remains in conformation I, with a transient change to conformation II. When Asp_CAT_ is protonated, Ile257 adopts an orientation similar to conformation II within 10 ns of the simulation and maintains it throughout the remaining 40 ns of the MD run. This change to conformation II is enabled by the reorientation of His_CAT_, which creates sufficient space to facilitate the entry and exchange of water and to allow the Ile257 side chain to rotate into the vicinity of the T2Cu without steric hindrance. The MD simulation shows that the average distance between the T2Cu and the CD1 atom of Ile257 shortens by almost ∼2 Å compared with conformation I. This result is consistent with the observed orientations of Ile257 in the 240 K crystal structures. Moreover, the MD suggests that some reorientation of His_CAT_ is required to enable the transition of Ile257 to conformation II.

The highly conserved Ile_CAT_ residue in the CuNiRs is thought to play an important role in catalysis by enforcing a bidentate O-binding mode for nitrite at the T2Cu that establishes critical hydrogen bonding to Asp_CAT_ (Boulanger & Murphy, 2003 ▸). Nitrite binding and turnover is a dynamic activity and the flexibility of Ile257 observed here may be significant in this process by imposing the steric constraints required for optimizing the nitrite-binding geometry. The dynamic behaviour of the capping residue may also be important for establishing the geometry of the catalytic product, NO, which has always been observed in crystals bound to the T2Cu in a side-on rather than an end-on mode (see, for example, Antonyuk *et al.*, 2005 ▸; Tocheva *et al.*, 2007 ▸), including where NO was generated *in situ* through a 100 K MSOX series (Horrell *et al.*, 2016 ▸). Conformation II of Ile257 compresses the T2Cu pocket more than conformation I and would tend to further prohibit end-on formation of NO. Quantum chemical calculations have instead suggested a preference for end-on binding in the solution state, where increased flexibility of the active-site residues Asp_CAT_ and Ile_CAT_ may relax hydrogen-bonding and steric constraints, allowing NO to adopt the end-on orientation (Solomon *et al.*, 2014 ▸). The MD simulations reveal the symbiotic dynamics concerning His_CAT_, Asp_CAT_ and Ile_CAT_ that are relevant to active-site solvation, ligand binding and catalysis in the CuNiRs.

## Supplementary Material

PDB reference: *Achromobacter cycloclastes* nitrite reductase, data set ds1_240K_, 5n8f

PDB reference: data set ds2_240K_, 5n8g

PDB reference: data set ds3_240K_, 5n8h

PDB reference: data set ds_10K_, 5n8i

Supporting figures, structure and molecular-dynamics simulations.. DOI: 10.1107/S2052252517007527/be5278sup1.pdf

## Figures and Tables

**Table 1:** Crystallographic data collection and structure refinement for wild-type *Ac*NiR Statistics are shown for three consecutive data sets measured from one crystal at 240 K and a single data set for one crystal (grown in the same batch) at 100 K. Values in parentheses are for the highest resolution shell. Data were processed using CC_1/2_ ≥ 0.5 and *I*/σ(*I*) ≥ 1.0 (outer shell) cutoffs.

	ds1_240K_	ds2_240K_	ds3_240K_	ds_100K_
Structure				
Unit-cell parameter (Å)	96.1	96.1	96.2	94.8
Resolution (Å)	29.0–1.38	29.0–1.47	29.0–1.65	47.4–1.40
Unique reflections	59054 (2062)	50265 (2473)	35669 (1910)	54929 (2667)
Multiplicity	4.1 (1.7)	4.4 (3.4)	4.5 (4.3)	3.2 (3.5)
*R*_p.i.m._ (%)	6.7 (52.9)	6.5 (50.2)	6.1 (57.3)	5.5 (50.2)
*R*_meas_ (%)	14.0 (82.6)	13.7 (94.0)	13.2 (101.0)	8.4 (82.6)
CC_1/2_ (outer shell)	0.53	0.51	0.50	0.51
〈*I*/σ(*I*)〉	7.0 (1.1)	7.7 (1.5)	9.2 (1.4)	10.9 (1.5)
Completeness (%)	97.1 (69.2)	99.5 (98.9)	99.4 (99.8)	98.4 (97.7)
Wilson *B* factor (Å^2^)	9.4	11.5	15.9	9.1
Refinement				
*R*_work_/*R*_free_ (%)	11.8/15.7†	14.6/16.5	14.4/17.0	15.3/18.1
R.m.s.d., bond lengths (Å)	0.014	0.013	0.012	0.013
R.m.s.d., bond angles (°)	1.74	1.66	1.58	1.72
ML-based ESU (Å)	0.037	0.045	0.065	0.046
Average protein *B* factor (Å^2^)	11.7	14.1	19.0	13.3
Average water *B* factor (Å^2^)	25.9	26.6	31.3	24.2
Ramachandran plot (No. of residues)				
Favoured regions	327	328	327	327
Allowed regions	4	3	4	5
Cumulative absorbed dose (MGy)	0.1	0.3	0.5	0.03
PDB code	5n8f	5n8g	5n8h	5n8i

† Anisotropic refinement.
