# Supplementary material for: Active-site protein dynamics and solvent accessibility in native Achromobacter cycloclastes copper nitrite reductase
Source: IUCrJ. 2017 Jun 16;4(Pt 4):495–505. doi: 10.1107/S2052252517007527 (PMC5571812; doi:10.1107/S2052252517007527)
Supplement: Supplementary file 1 [file m-04-00495-sup1.pdf]

IUCrJ

Volume 4 (2017)

Supporting information for article:

Active-site protein dynamics and solvent accessibility in native *Achromobacter cycloclastes* copper nitrite reductase

Kakali Sen, Sam Horrell, Demet Kekilli, Chin W. Yong, Thomas Keal, Hakan Atakisi, David W. Moreau, Robert E. Thorne, Michael A. Hough and Richard W. Strange

S1. AcNiR crystal structure at 100 K

Figure S1 shows the T2Cu coordination sphere of the 100 K crystal structure $ds1_{100K}$, described in the main text.

S2. Molecular dynamics of alternative protonation states of Asp_{CAT} and His_{CAT}.

At pH 5, His_{CAT}, one of the active participant in proton transfer, is expected to be protonated. The Asp_{CAT} sidechain would be expected to have a pK_a of 3.9, however, depending on the instantaneous pH of the active site, it could be protonated or deprotonated in our simulations. In the deprotonated state of Asp_{CAT}, instantaneous proton transfer from His_{CAT} to Asp_{CAT} via the bound water molecule cannot be ruled out. Thus, it leads us to investigate all the possible protonation states of these residues. The two most likely situations, Asp_{CAT} and His_{CAT} both protonated (Asp98p) and only His_{CAT} protonated (Asp98) as proposed by Solomon *et. al.* corresponding to low and high pH cases, respectively, are discussed in the main text. The remaining four possible protonation states of the two residues are: (i) Asp98 (deprotonated Asp) – HSD (H on Nδ of His255), (ii) Asp98 - HSE (H on Nε of His255), (iii) Asp98p (protonated Asp) – HSD, (iv) Asp98p – HSE. These systems are referred to henceforth as 'Asp98-HSD, 'Asp98-HSE', 'Asp98p-HSD' and 'Asp98p-HSE'. The MD setup is same as that described in the main text, except the Asp98-HSD, Asp98-HSE systems were electroneutral and required no additional Cl^- ions, whereas electroneutrality of Asp98p-HSD and Asp98-HSE systems required addition of 3 Cl^- ions in the system.

As described in the main text, the centres of mass of Asp_{CAT} and T2Cu are used to measure of the proximal and gatekeeper positions of Asp_{CAT}, while the number of water molecules within 3 Å of the T2Cu site provide a measure of the accessibility of water at the T2Cu site. Figure S3 shows the T2Cu-Asp_{CAT} COM separations. In all cases when Asp_{CAT} is deprotonated, the proximal orientation is preferred for both of the Asp-HSD and Asp-HSE systems and the average T2Cu-Asp_{CAT} distance agrees well with that observed in crystal structures (4 Å). When Asp_{CAT} is protonated, it flips between proximal and gatekeeper positions, a behaviour similar to MD simulations of the Asp98p system described in the main text.

The water accessibility at the T2Cu site for MD simulations using different states of His_{CAT} for deprotonated and protonated Asp_{CAT} systems resembles that of Asp98 and Asp98p (main text), respectively (fig S4-6). There is very slow or almost no exchange of the coordinated water in 2 chains of Asp98-HSE system, consistent with the proximal orientation of Asp_{CAT}. There is much faster exchange of water and higher solvent accessibility for the Asp98p-HSD and Asp98p-HSE systems, which corroborates with the alternative conformations attainable by Asp_{CAT} on protonation.

For both the Asp98p-HSD and Asp98p-HSE systems the deviations of the three hydrophobic residues in the immediate vicinity of the water channel at the T2Cu site (Val142, Ala137 and Ile257) are less significant than the movement of Asp_{CAT} or His_{CAT}. This is consistent with the finding described in the main text that the increase in number and throughput of exchangeable water molecules occupying the active site pocket in protonated Asp_{CAT} is triggered by the switch of the Asp98p sidechain from the proximal to the gatekeeper position, a movement that does not occur during the same time-scale for the deprotonated Asp98 (figure S7-S8).

Figure S9-10 show the water structure (within 3 Å) and close neighbour protein residues of the His_{CAT} residue, corresponding to figure 3 of the main text

For all the deprotonated Asp_{CAT} systems, Asp_{CAT} maintains its proximal position, along with the Asp_{CAT}- His_{CAT} separation (figure S11), while for the protonated Asp_{CAT} systems, a more complicated picture emerges (figure S12).

In case of Asp98p-HSD simulations, the Asp_{CAT} switches from proximal to gatekeeper positions while His_{CAT} is maintained throughout at its crystallographic position in all the monomeric chains (figure S12, see right panel). This restricted dynamic of His_{CAT} residue could be outcome of a stable hydrogen bond between Hδ and the backbone oxygen atom of Glu279. The dynamics of His_{CAT} in the Asp98p-HSE system matches with that of Asp98p, where the His_{CAT} deviates from its crystallographic position in two of the three chains. The dynamics of His_{CAT} is correlated to the movement of Ile257. Displacement of His_{CAT} from its crystallographic position, provides room for Ile257 to move closer to T2Cu site, adopting conformation *II* (figure S13). The absence of such His_{CAT} dynamics in deprotonated Asp98 (main text), Asp98-HSD, Asp98p-HSD and Asp98-HSE systems, restricts Ile257 predominantly to conformation *I* (in chain B of Asp98-HSE a transient occurrence of conformer *II* is observed).

S3. Molecular dynamics based on crystal structure with two T2Cu coordinated waters

To investigate the behaviour at pH 5 (when His_{CAT} is assumed protonated, as in the main text) and to explore water accessibility at the T2Cu site in the presence of two bound waters, the Asp98p (protonated) and Asp98 (deprotonated) states were again chosen and were subjected to MD simulations. The one water coordinated to T2Cu was replaced by two water molecules from the 240 K crystal structures. This was possible as the overall structures are similar at cryogenic and higher temperatures. The system setup followed the same protocols given in the main text, with a production run to 30 ns.

The results were entirely consistent with the MD simulations using one coordinated water molecule, described in the main text, implying again that the local perturbation in the electrostatics is

the guiding factor for the water accessibilities and conformational changes of the catalytically important residues. Details of these additional calculations are shown in figures S14-S19.

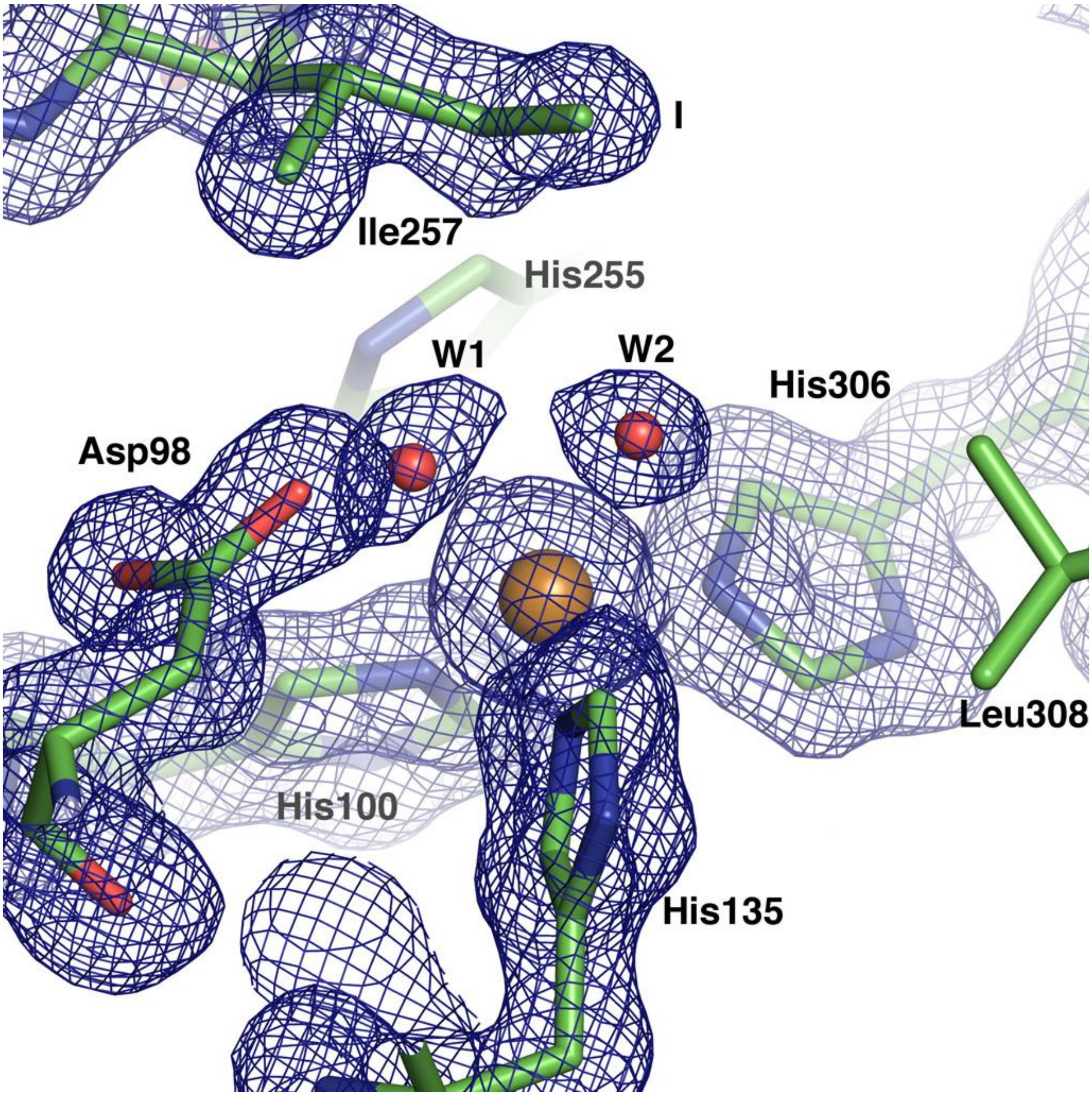

Figure S1 The T2Cu site of wild-type *Ac*NiR at 100 K, showing two bound water molecules and a single Ile257 orientation (conformation *I*). The $2F_o$-F_c electron density map is contoured at 0.52 e/$Å^3$.

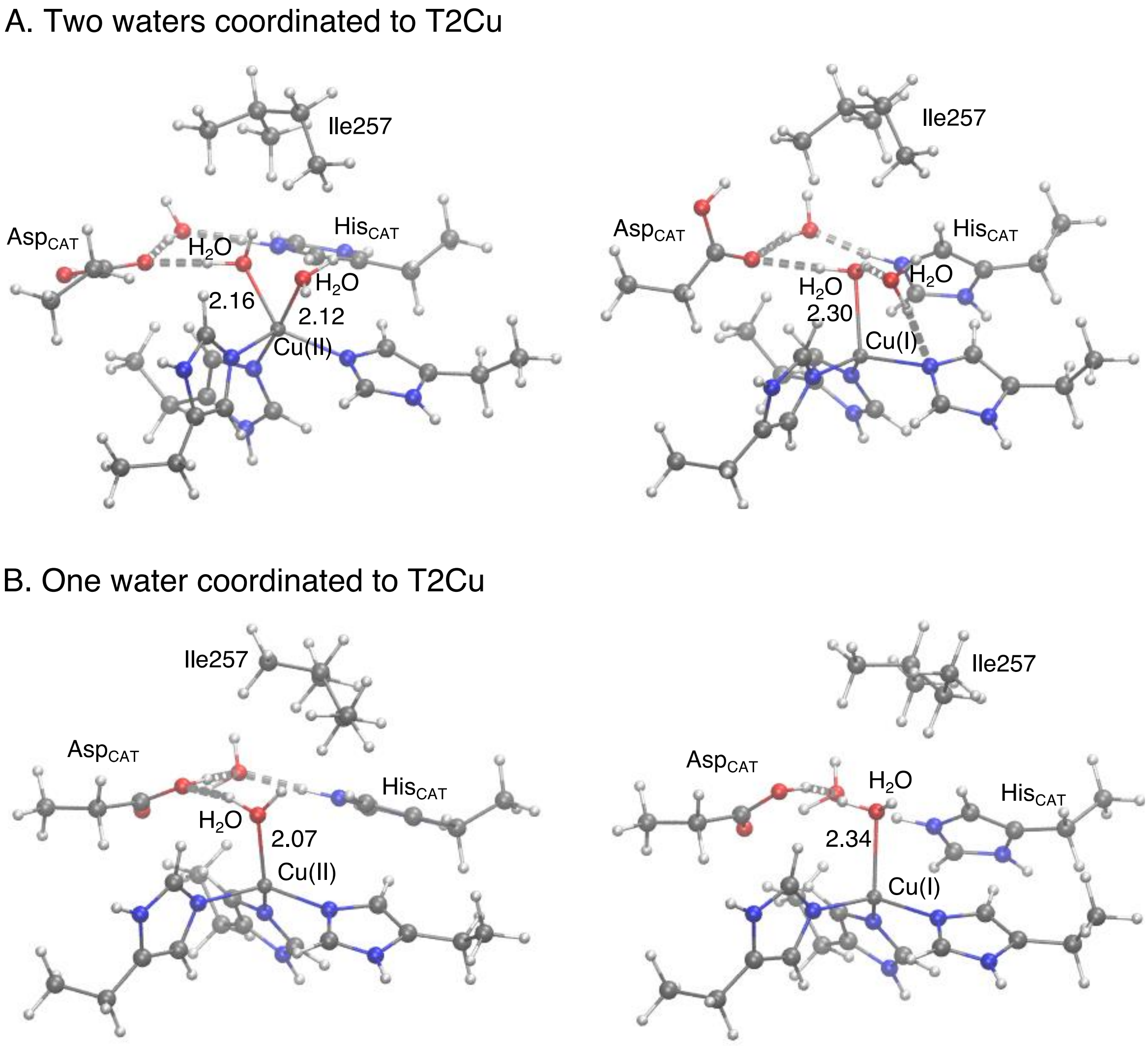

Figure S2 DFT optimized structures of the T2Cu site with different oxidation states with Asp$_{CAT}$ protonated on the oxygen atom close to T2Cu, modelled from crystal structures obtained at 240 K. (a) Oxidised state (left panel) showing two waters coordinated, and the reduced state (right panel) with one water lost from the coordination sphere; (b) Oxidised state (left panel) with one water coordinated, and the reduced state (right panel) in which this water is retained with an increased bond length. Both Asp$_{CAT}$ and His$_{CAT}$ are protonated and distances are given in Å.

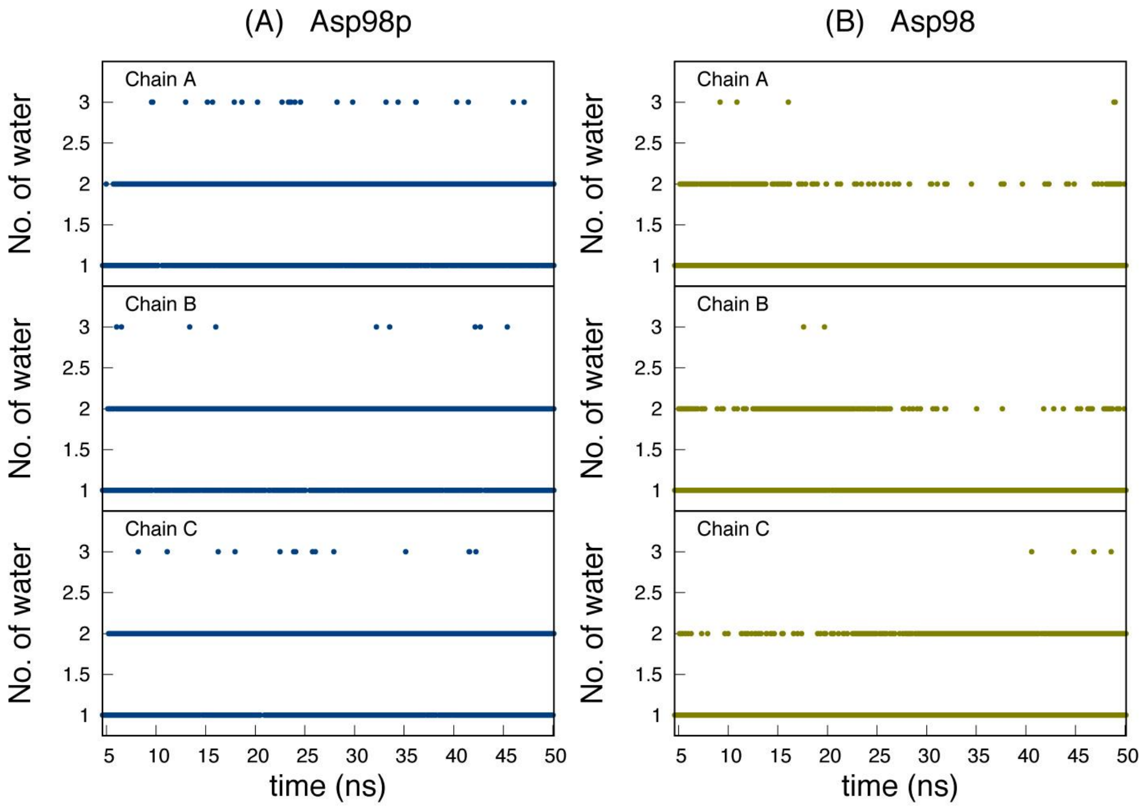

Figure S3 Total count of water molecules within 3Å of T2Cu site for Asp98 (right panel) and Asp98p (left panel) systems. Each monomeric unit is shown separately and the average number of among all the monomers reported in the text

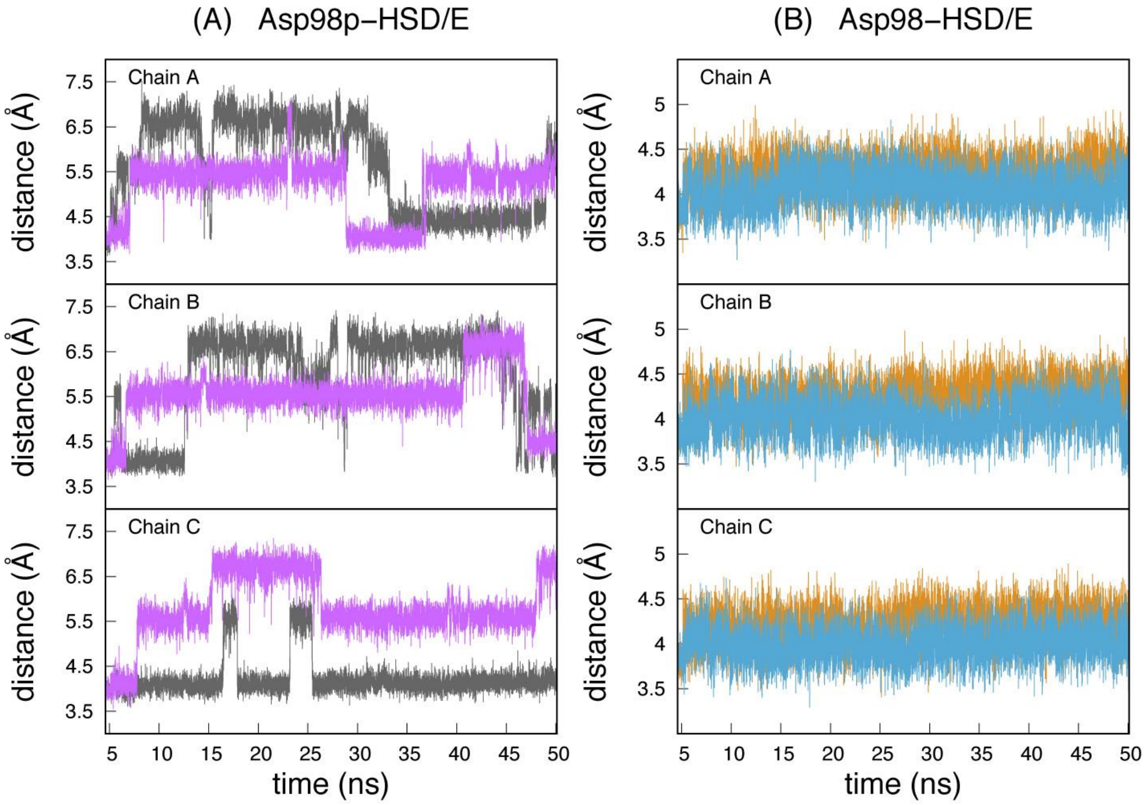

Figure S4 Cu-Asp$_{CAT}$ distances for different histidine protonation states during a 50 ns MD simulation. The left panel provides Cu-Asp$_{CAT}$ distances for Asp98-HSD (magenta) and Asp98-HSE (gray) states, with a maximum separation of T2Cu-Asp$_{CAT}$ of 6.9 +/- 0.2 Å for both cases; the right panel provides the same information for Asp98p-HSD (teal) and Asp98p-HSE (orange) states, with an average separation of 4.2 +/- 0.2 and 4.0 +/- 0.2 Å respectively, similar to the crystal structure values.

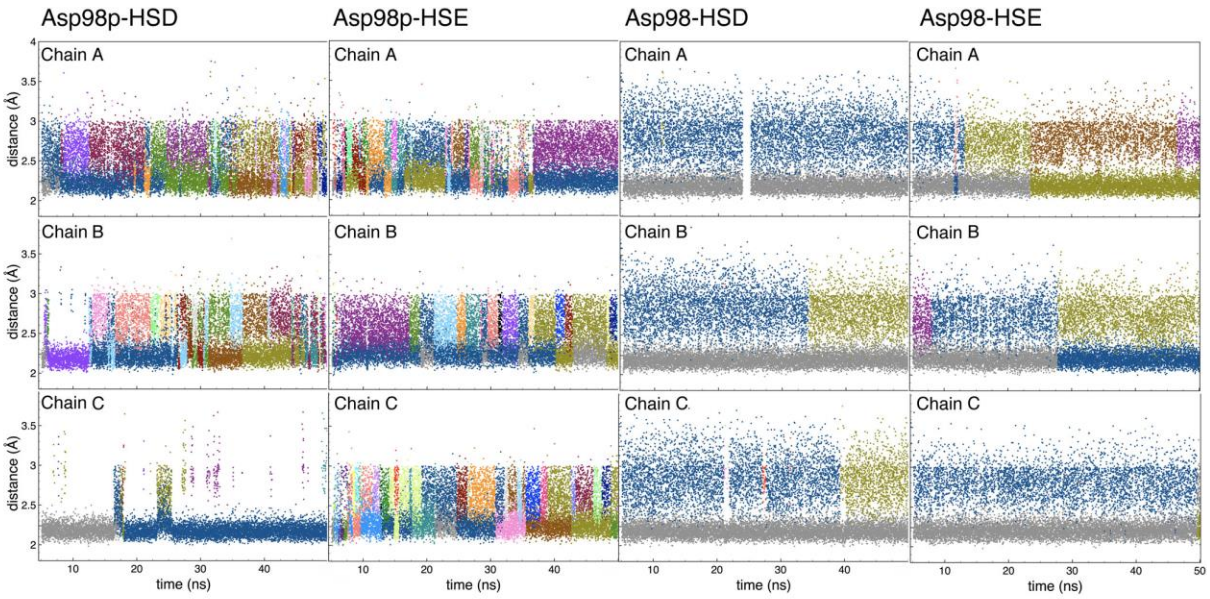

Figure S5 Water accessibility for the two protonation states of Asp_{CAT} with deprotonated HSE and HSD states of His_{CAT}. Water molecules found in MD simulations within 3 Å of the T2Cu atom in each monomer (chains A, B and C) of the *Ac*NiR trimer are shown in different colours, with the bound water in the original crystal structure shown in gray. Two or more water molecules were found in each monomer of Asp98-HSD and Asp98-HSE sytems for 69 +/- 2 %, and 59 +/- 5 %, respectively during the MD simulations; these values are 79 +/- 4 % for Asp98p-HSE and 51 +/- 28 % (lower fraction of water in chain C) for Asp98p-HSD.

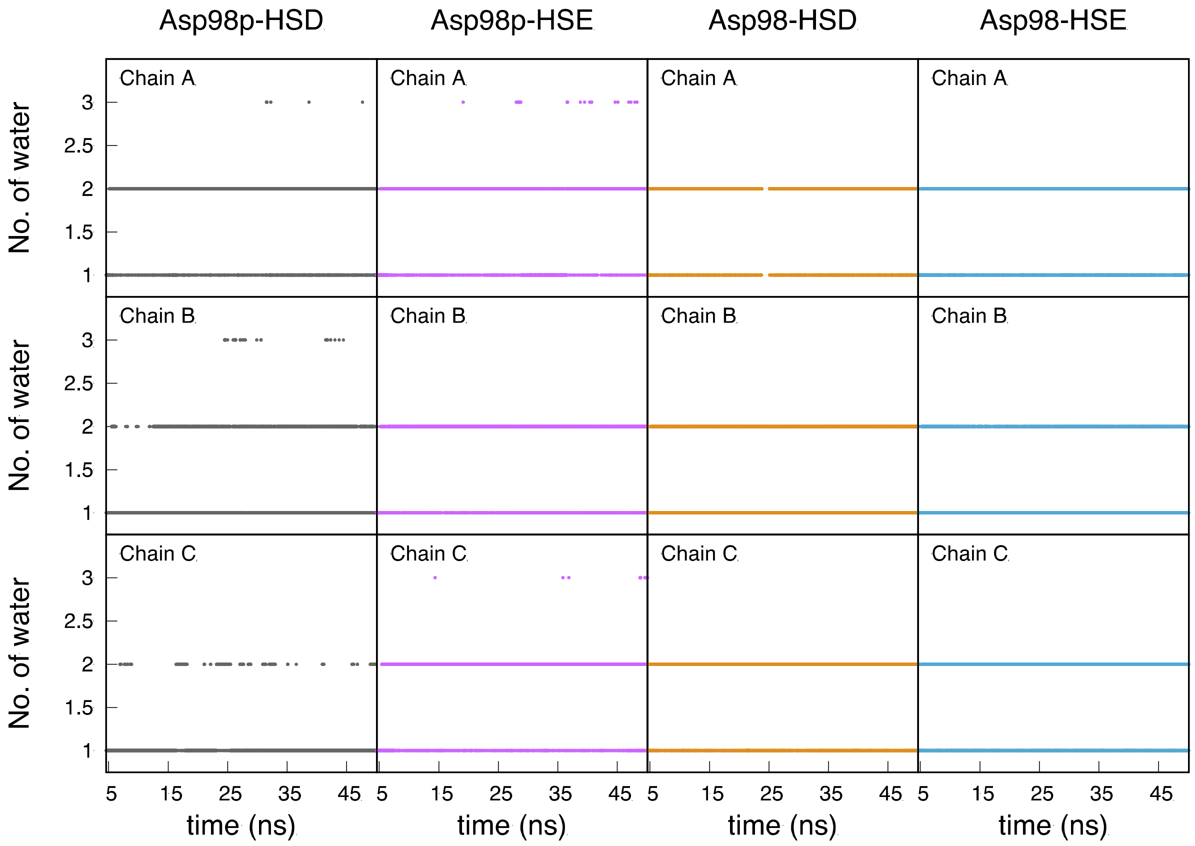

Figure S6 Total number of waters within 3Å of T2Cu site for different protonation states of Asp_{CAT} and HSE /HSD states of His_{CAT}

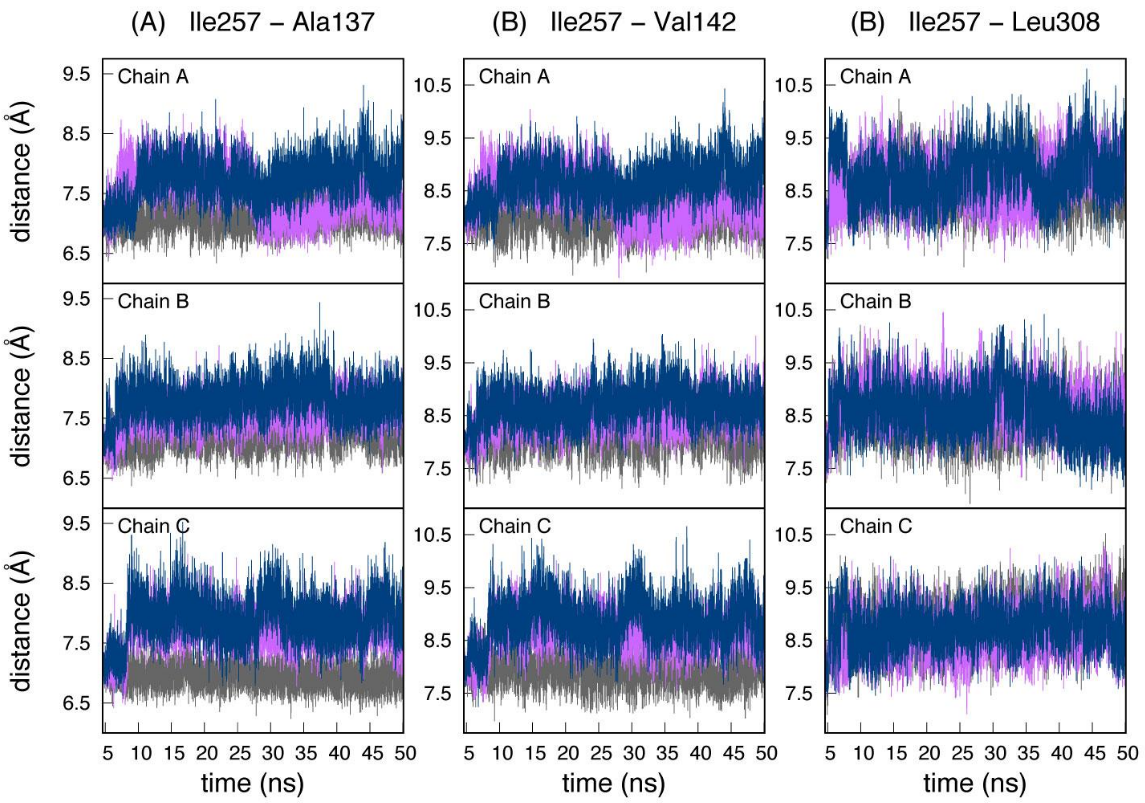

Figure S7 Distance (centre of mass) evolution among the hydrophobic residues (Ile127, Ala137, Val142 and Leu308) that define the active site solvent channel for Asp98/HSP (gray), Asp98/HSD (magenta) and Asp98/HSE (blue)

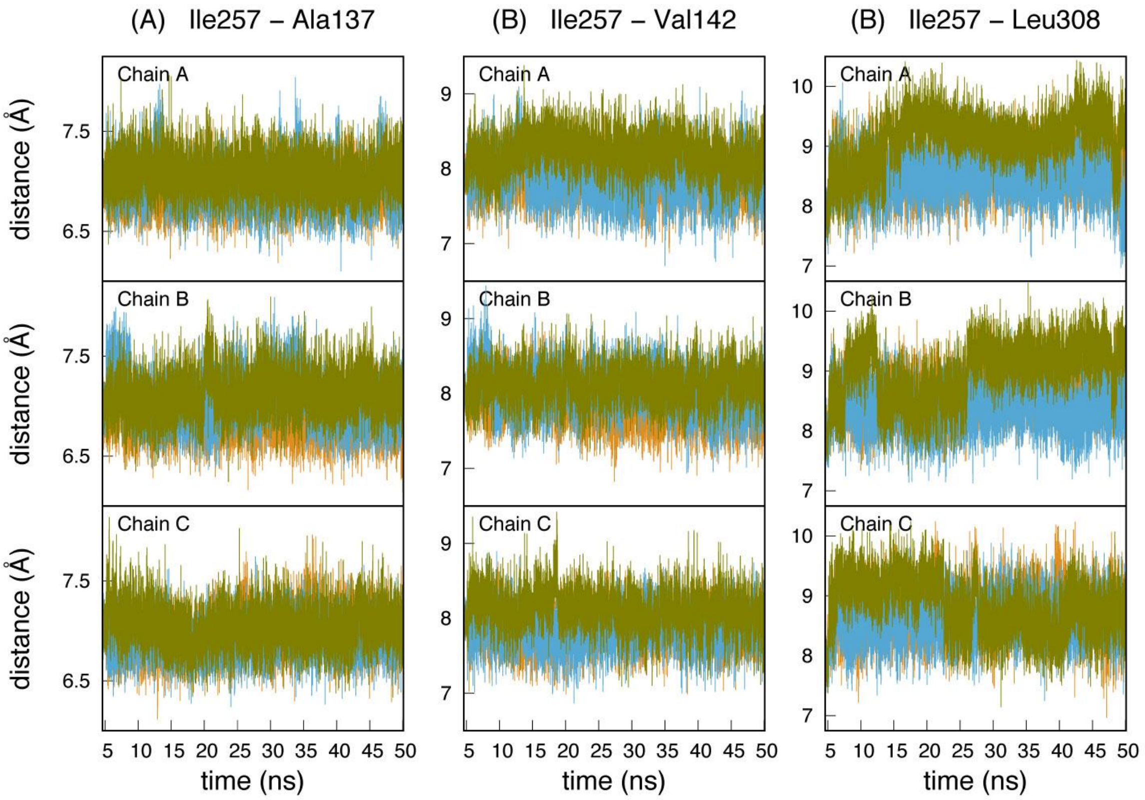

Figure S8 Distance (centre of mass) evolution between Ile257 and the hydrophobic residues Ala137, Val142 and Leu308 that define the active site solvent channel for states with Asp98p/HSP (green), Asp98p/HSD (orange) and Asp98p/HSE (teal)

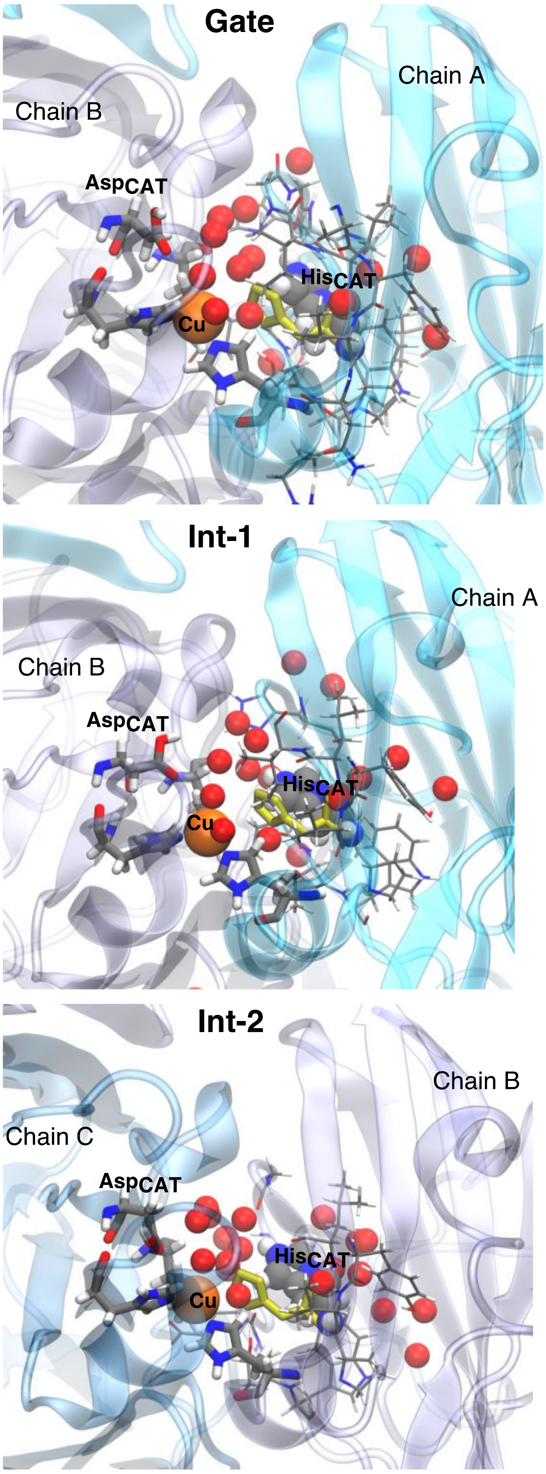

Distances between His_{CAT} and nearest residues (protein and water) in Gate conformation

His_{CAT} **O**	Val304 **N**	3.32 Å
His_{CAT} **N**	Val304 **O**	3.44 Å
His_{CAT} **ND1**	TRP281 **CZ3**	3.40 Å
His_{CAT} **NE2**	WAT249 **OH2**	2.75 Å

Distances between His_{CAT} and nearest residues (protein and water) in Int-1 conformation

His_{CAT} **O**	Val304 **N**	3.26 Å
His_{CAT} **N**	Val304 **O**	3.17 Å
His_{CAT} **CB**	Thr280 **CA**	3.89 Å
His_{CAT} **NE2**	Glu279 **CA**	3.31 Å
His_{CAT} **ND1**	WAT475 **OH2**	2.73 Å
His_{CAT} **NE2**	WAT450 **OH2**	2.69 Å

Distances between His_{CAT} and nearest residues (protein and water) in Int-2 conformation

His_{CAT} **O**	Val304 **N**	3.30 Å
His_{CAT} **N**	Val304 **O**	3.04 Å
His_{CAT} **NE2**	Gly259 **CA**	3.33 Å
His_{CAT} **CB**	TRP281 **CZ3**	3.82 Å
His_{CAT} **ND1**	WAT7517 **OH2**	3.06 Å
His_{CAT} **NE2**	WAT5067 **OH2**	2.79 Å
His_{CAT} **CE1**	WAT7122 **OH2**	2.94 Å
His_{CAT} **CD2**	WAT299 **OH2**	3.56 Å

Figure S9 Immediate protein (3Å) and water residues surround the displaced His_{CAT} residue in Asp98p system for the gate, the Int-1 and the Int-2 conformations of Asp_{CAT} corresponding to the same given in figure 3 of the main text. In yellow is the position of His_{CAT} residue in the crystal structure.The distances of the closest residues (protein and water) to the displaced His_{CAT} are provided in the corresponding tables on the right

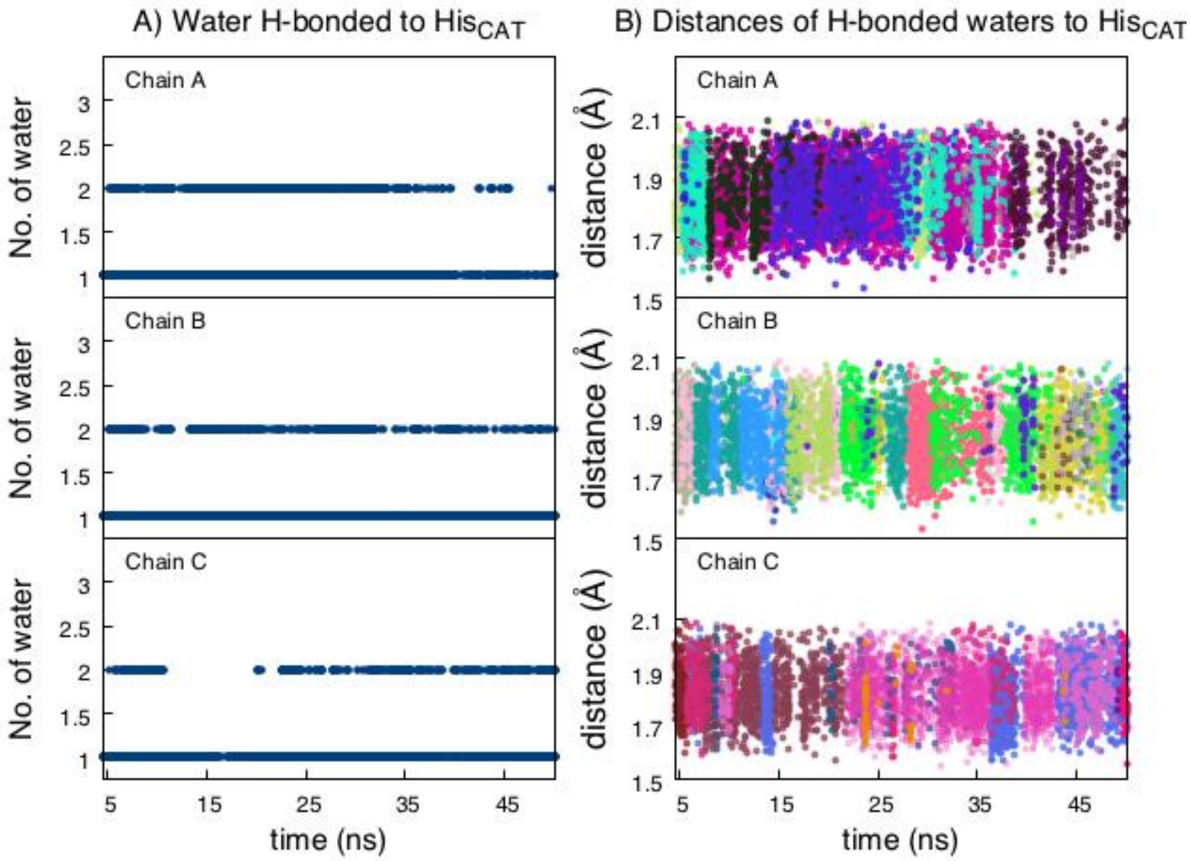

Figure S10 A) Total number of waters within 3 Å of the His_{CAT} side chain in the Asp98p system. B) The distances of water molecules found in MD simulations within 3 Å of the side chain atoms ND1, HD1, NE2 and HE2 of displaced His_{CAT} in each monomer (chains A, B and C), shown in different colours

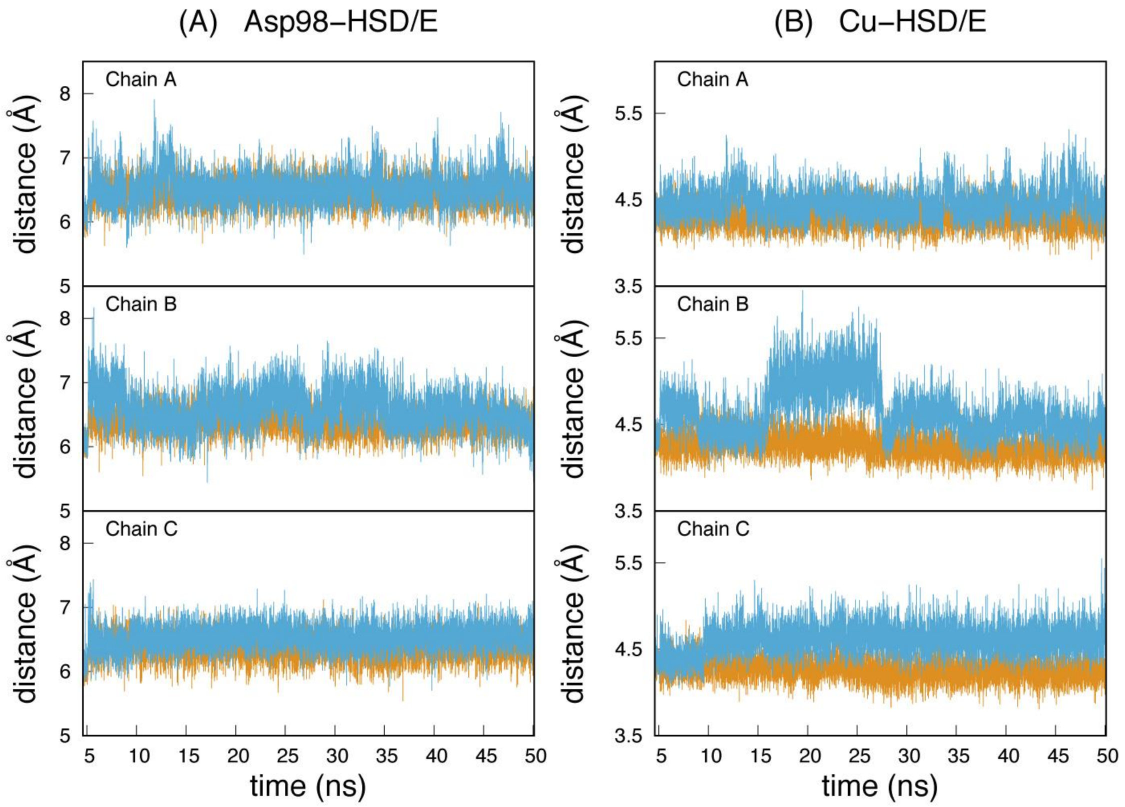

Figure S11 The time evolution of the centre of mass Asp_{CAT}-His_{CAT} and Cu-His_{CAT} separations for deprotonated active site states. Asp98-HSD (orange) and Asp98-HSE (teal).

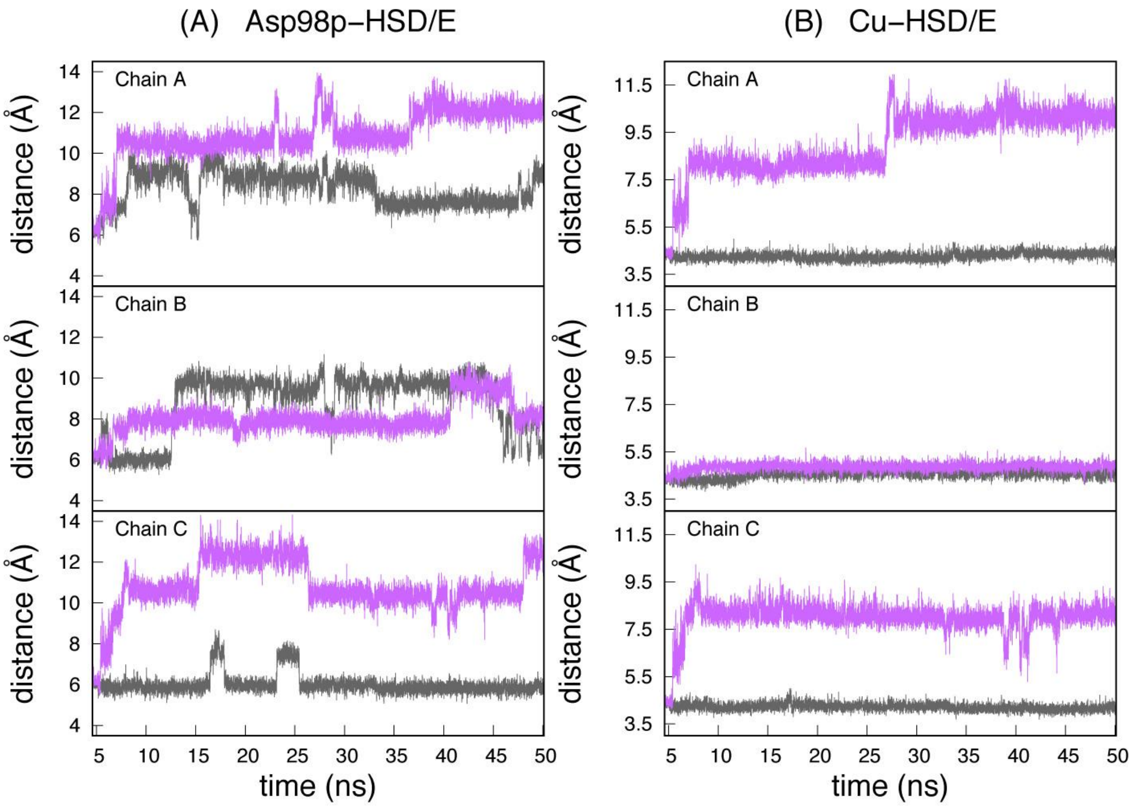

Figure S12 The time evolution of the centre of mass Asp_{CAT}-His_{CAT} and Cu-His_{CAT} separations for protonated Asp_{CAT} states. Asp98p-HSD (gray) and Asp98p-HSE (magenta). The right hand panel shows that the His_{CAT} in HSD state is kept in the same position observed in the crystal structures, while for the HSE state the His_{CAT} is moves away from the T2Cu, behaviour that is similar to that seen for MD simulations of protonates His_{CAT} (main text).

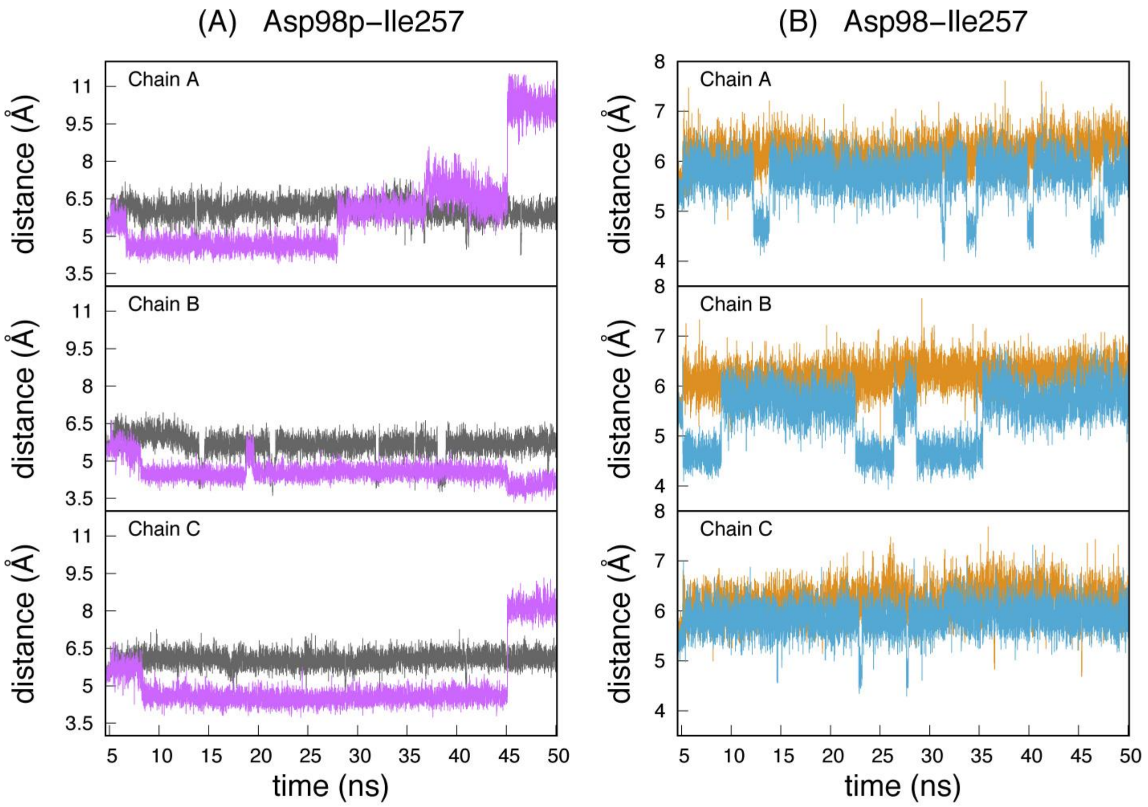

Figure S13 Time evolution of distance between the T2Cu atom and the Ile257 CD atom. MD simulations are shown for protonated (a) and deprotonated (b) states of $Asp_{CAT.}$ The variants are Asp98/HSD (orange), Asp98/HSE (teal), Asp98p/HSD (gray) and Asp98p/HSE (magenta).

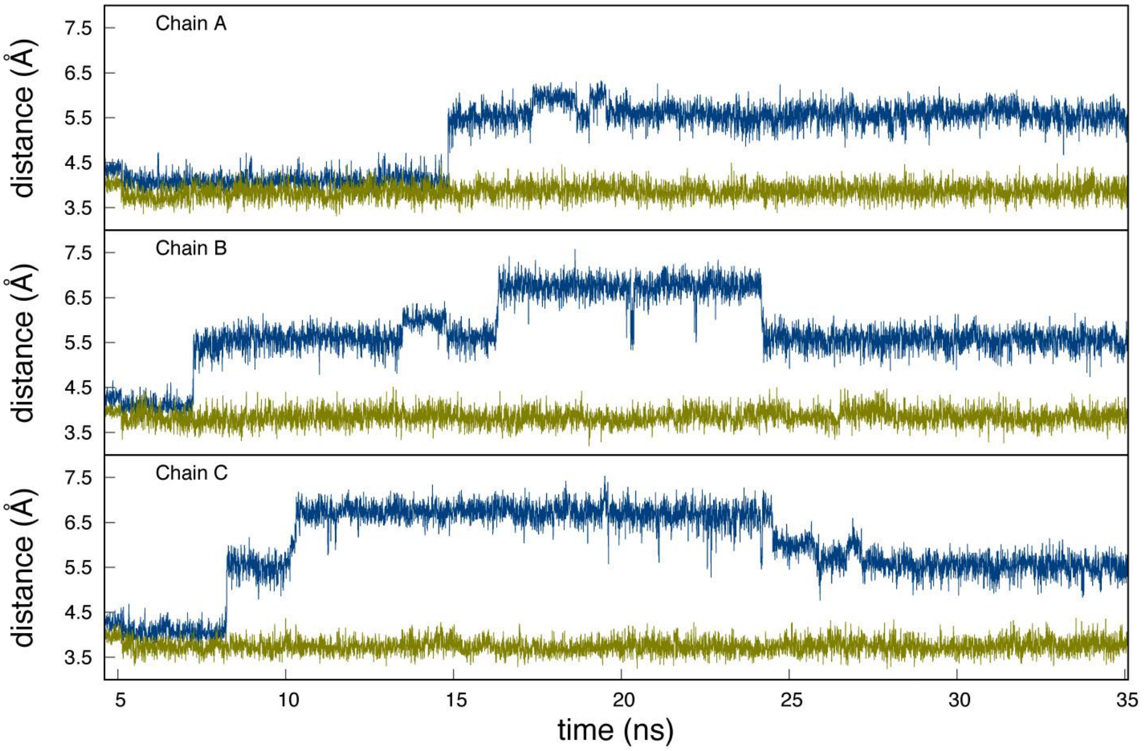

Figure S14 The variation in T2Cu-Asp98p (blue) and T2Cu-Asp98 (green) distances using a starting model for the MD based on a wild-type *Ac*NiR crystal structure (this work) with 2 water molecules coordinated to the T2Cu.

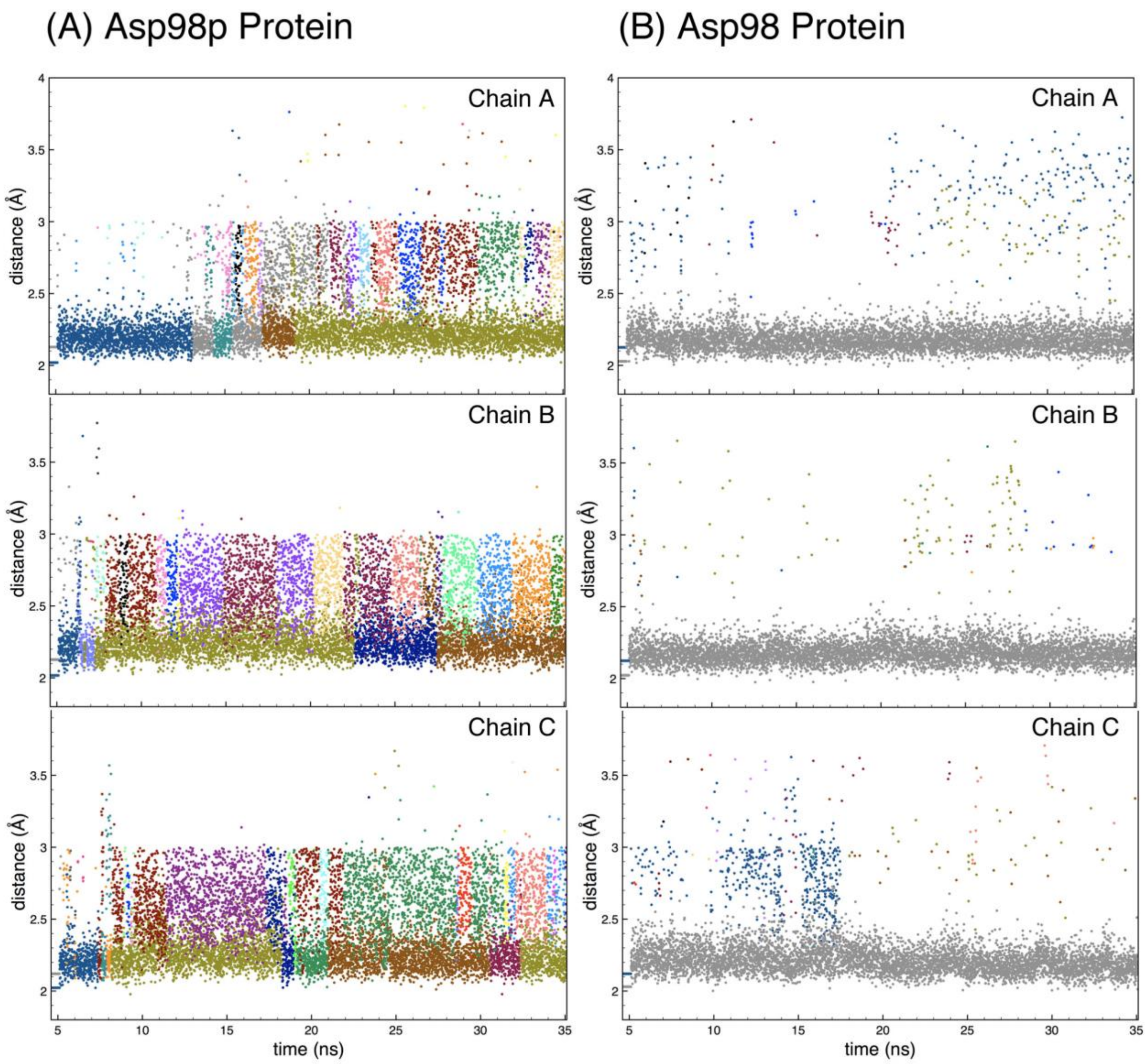

Figure S15 Water accessibility within 3Å of the T2Cu using a starting structure for MD with 2 waters coordinated to T2Cu.The protonated (a) and deprotonated (b) states of Asp_{CAT} are shown. Water accessibility and exchange are facilitated with protonation: in 65.4 +/- 14.2 % of time there are two or more waters in Asp98p-HSP which reduces to 5.6 +/- 2.9 % in Asp98-HSP

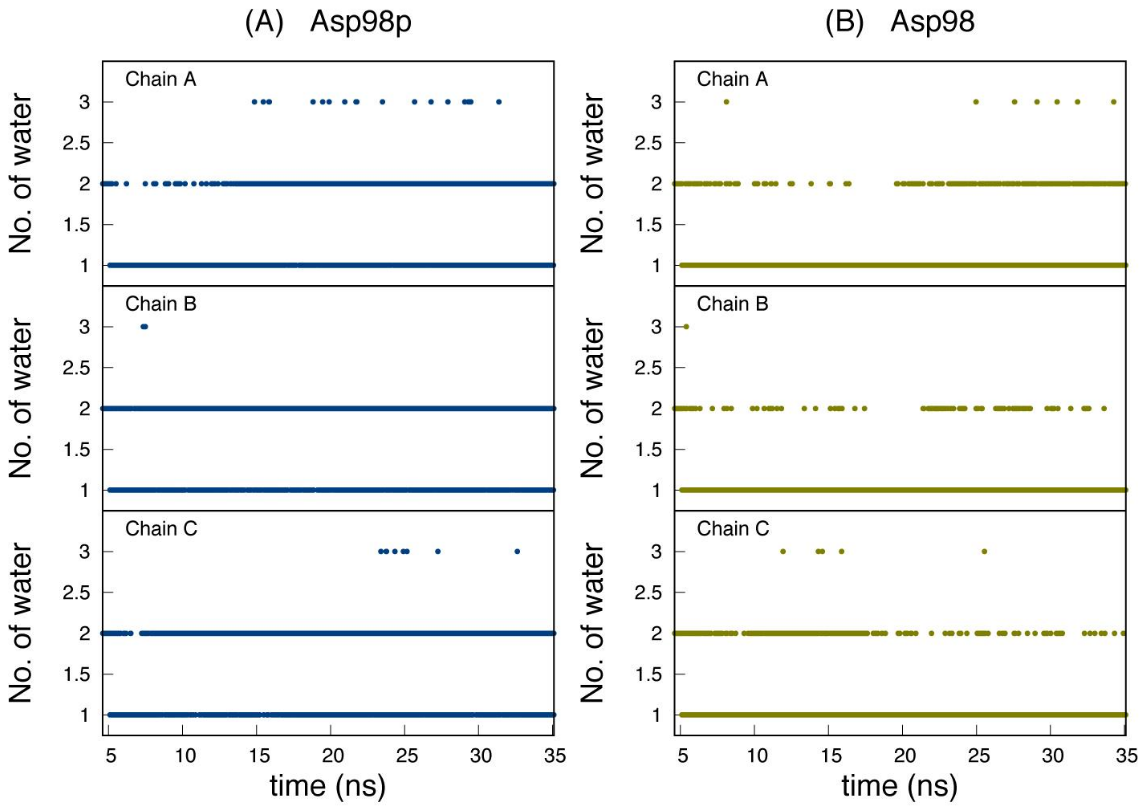

Figure S16 Total number of water within 3A of T2Cu using a starting structure for MD with 2 waters coordinated to T2Cu. The protonated Asp_{CAT} states (a) Asp98p (blue) and deprotonated (b) Asp98 (green) are shown.

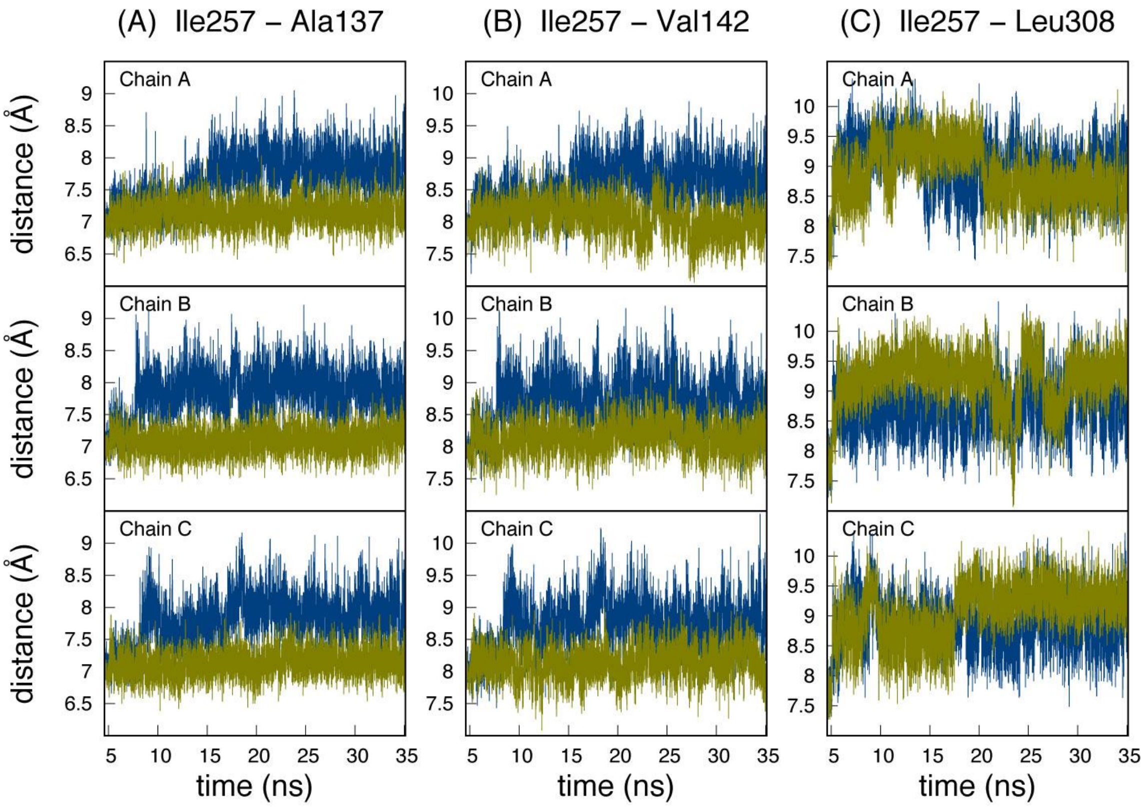

Figure S17 Distance evolution between Ile257 and the hydrophobic residues Ala137, Val142 and Leu308 that define the active site solvent channel, using a starting structure for MD with 2 waters coordinated to T2Cu, for states with protonated His_{CAT} and Asp98 (green) and Asp98p (blue).

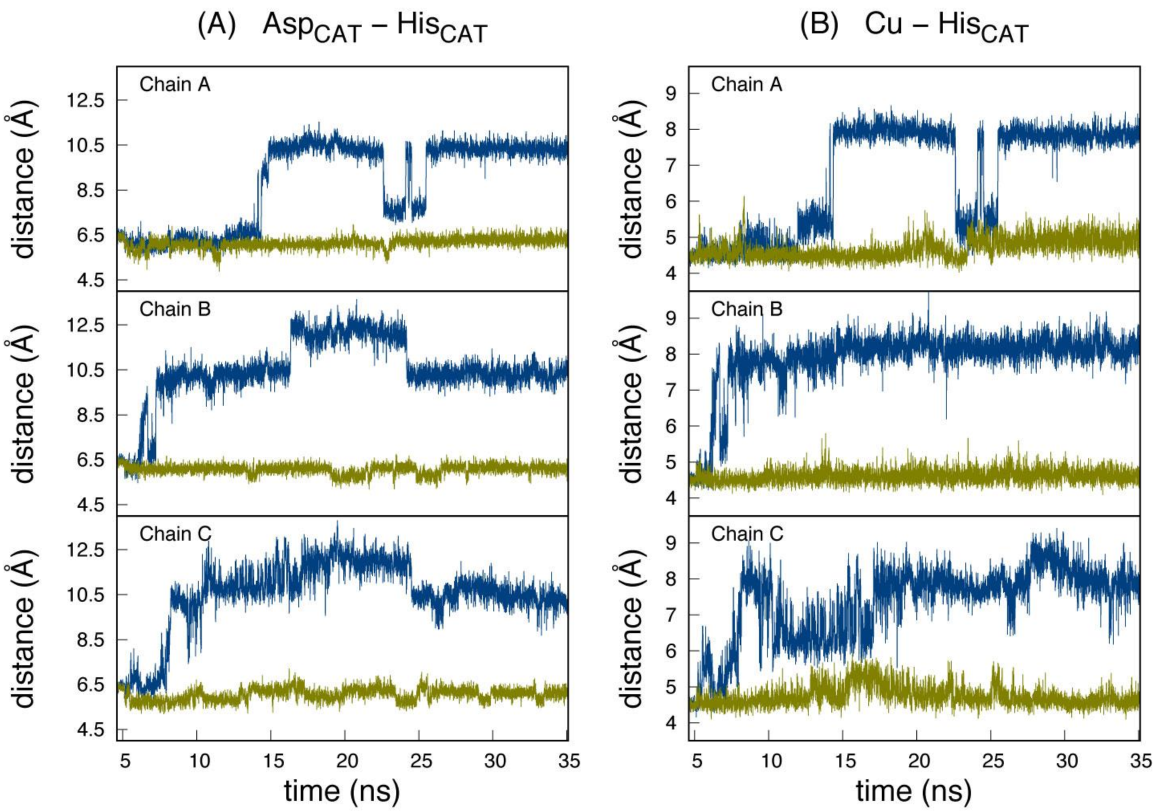

Figure S18 Time evolution of His$_{CAT}$ residue in each monomer of the *Ac*NiR trimer during MD simulations using a starting structure for MD with 2 waters coordinated to T2Cu. (a) Distance between centre of mass of His$_{CAT}$ ring atoms (heavy atoms only) and centre of mass of Asp98p carboxylic group (blue) and that Asp98 (green) states; (b) Distance between centre of mass of His$_{CAT}$ atoms and T2Cu.

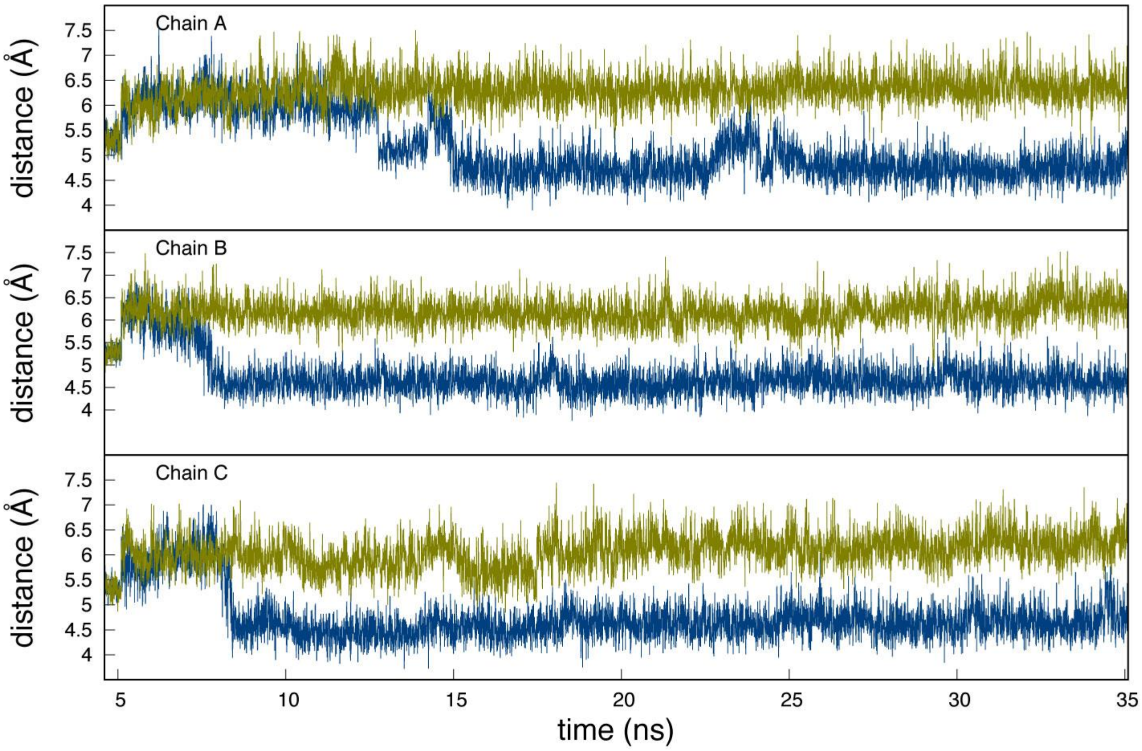

Figure S19 Time evolution of Ile257 residue in each monomer of the *Ac*NiR trimer during MD simulations using 2 waters initially coordinated to T2Cu. The distance between the sterically important Ile257 residue sidechain CD1 atom and the T2Cu atom is shown for the protonated Asp98p (blue) and deprotonated Asp98 (green) states of the protein.
